# Designer proteins that competitively inhibit Gα_q_ by targeting its effector site

**DOI:** 10.1016/j.jbc.2021.101348

**Published:** 2021-10-27

**Authors:** Mahmud Hussain, Matthew C. Cummins, Stuart Endo-Streeter, John Sondek, Brian Kuhlman

**Affiliations:** 1 Department of Biochemistry and Biophysics, University of North Carolina, Chapel Hill, North Carolina, USA; 2 Department of Pharmacology, University of North Carolina, Chapel Hill, North Carolina, USA; 3 Lineberger Comprehensive Cancer Center, University of North Carolina, Chapel Hill, North Carolina, USA

**Keywords:** heterotrimeric G protein, Gα_q_, Rosetta molecular modeling program, protein design, cancer, molecular modeling, phospholipase C, peptide interaction, CD, circular dichroism, FACS, fluorescent-activated cell sorting, GAP, GTPase-activating protein, GPCR, G-protein-coupled receptor, HTH, helix-turn-helix, PLC-β, phospholipase C-β, SRE, serum response element

## Abstract

During signal transduction, the G protein, Gα_q_, binds and activates phospholipase C-β isozymes. Several diseases have been shown to manifest upon constitutively activating mutation of Gα_q_, such as uveal melanoma. Therefore, methods are needed to directly inhibit Gα_q_. Previously, we demonstrated that a peptide derived from a helix-turn-helix (HTH) region of PLC-β3 (residues 852–878) binds Gα_q_ with low micromolar affinity and inhibits Gα_q_ by competing with full-length PLC-β isozymes for binding. Since the HTH peptide is unstructured in the absence of Gα_q_, we hypothesized that embedding the HTH in a folded protein might stabilize the binding-competent conformation and further improve the potency of inhibition. Using the molecular modeling software Rosetta, we searched the Protein Data Bank for proteins with similar HTH structures near their surface. The candidate proteins were computationally docked against Gα_q_, and their surfaces were redesigned to stabilize this interaction. We then used yeast surface display to affinity mature the designs. The most potent design bound Gα_q/i_ with high affinity *in vitro* (K_D_ = 18 nM) and inhibited activation of PLC-β isozymes in HEK293 cells. We anticipate that our genetically encoded inhibitor will help interrogate the role of Gα_q_ in healthy and disease model systems. Our work demonstrates that grafting interaction motifs into folded proteins is a powerful approach for generating inhibitors of protein–protein interactions.

Many extracellular hormones, neurotransmitters, and growth factors initiate a cellular response by binding and inducing conformational rearrangements in transmembrane G-protein-coupled receptors (GPCRs) (1, 2, 3). Consequently, activated GPCRs catalyze the exchange of GDP for GTP in the α subunit of heterotrimeric G proteins (Gαβγ) (4, 5). Upon binding GTP, the Gα subunit dissociates from the Gβγ subunits and engages downstream effectors, resulting in signal transduction and amplification (6). GTPase-activating proteins (GAPs) terminate signal transduction by binding Gα subunits and catalyzing the hydrolysis of GTP to GDP, returning the protein to its basal state (7, 8). Heterotrimeric G proteins are broadly classified into four families according to their Gα subunit: Gα_i_, Gα_s_, Gα_q_, and Gα_12/13_ (8, 9). Gα_i_ and Gα_s_ families regulate adenylyl cyclase; Gα_q_ members activate phospholipase C-β (PLC-β) isozymes and Trio-related RhoGEFs; and members of the Gα_12/13_ family activate p115RhoGEF and closely related exchange factors.

Approximately 30% of Food and Drug Administration–approved drugs target GPCRs (8, 10). In contrast, few molecules are available that directly inhibit Gα subunits. However, direct inhibitors for Gα subunits can be valuable tools for defining dependencies among intracellular signaling networks. For instance, pertussis and cholera toxins bind specifically to Gα_i_ and Gα_s_, respectively, and are routinely used to differentiate Gα_i_-mediated from Gα_s_-mediated signaling pathways (11, 12). Additionally, a recent report used a peptide derived from β-adrenergic receptor kinase 1 to uncover the role of Gα_q_ signaling in astrocytes (13).

Gα_q_ signaling is essential for various biological processes, including insulin-stimulated glucose transport, platelet aggregation, and vascular tone (14). Canonical effectors of Gα_q_ include PLC-β isozymes. Gα_q_ binds and activates PLC-β isozymes, which catalyze the hydrolysis of phosphatidylinositol 4,5-biphosphate to the second messengers, inositol 1,4,5-triphosphate, and diacylglycerol. Mutations that constitutively activate Gα_q_ drive ∼90% of all uveal melanomas (15, 16). Therefore, methods for directly inhibiting Gα_q_ are needed. However, current methods for direct inhibition of Gα_q_ are limited to natural products, such as the cyclic depsipeptide FR900359 and analogs and the aforementioned peptide from β-adrenergic receptor kinase 1 (9, 13, 17, 18, 19)^,^. These natural products require laborious and time-consuming purification from natural sources. Furthermore, their mechanism of action involves inhibiting the nucleotide exchange event of Gα_q_, thus preventing activation of Gα_q_. However, directly inhibiting the active form of Gα_q_ has the potential for greater efficacy of inhibition, particularly in uveal melanomas. Here, we engineer a protein that directly blocks activated Gα_q_ from binding downstream effectors.

A key structural feature in the interaction between Gα_q_ and PLC-β3 is the docking of a helix-turn-helix (HTH) from PLC-β3 into a broad depression on Gα_q_ (7). Loss of this interaction disrupts Gα_q_-mediated activation of PLC-β3. Peptides derived from this motif bind to Gα_q_ with low micromolar affinity and inhibit activation of PLC-β isozymes *in vitro* and in cells (20). Our collaborators used mRNA display to screen an extensive combinatorial library of HTH peptide variants and identified a mutation (V866W) at the peptide–protein interface that increases binding affinity (K_D_ = 290 nM, Fig. S1).

Here, we examine if binding affinity can be further increased by embedding the HTH in a folded protein. Circular dichroism (CD) experiments showed that the HTH peptide is unstructured in the unbound state (20), and therefore, there is likely to be a loss in conformational entropy upon binding Gα_q_. Thus, incorporating the HTH into a folded protein should allow preordering of the motif in a binding-competent conformation and provide a mechanism to increase binding affinity. To identify naturally occurring protein scaffolds well suited for stabilizing the HTH, we used the *MotifGraft* protocol in the Rosetta molecular modeling program (21, 22, 23). *MotifGraft* searches through high-resolution protein structures (“scaffolds”) for backbone segments with atomic coordinates that closely align with a structural motif of interest, in our case the HTH motif. The amino acid sequence of the motif is then threaded onto the aligned region of the scaffold, and surrounding residues in the scaffold are redesigned to accommodate the new sequence and desired binding interactions. We further optimized the binding motif and surrounding residues by performing affinity maturation with yeast display. Our best binder binds Gα_q_ with approximately 10-fold higher affinity than the HTH peptide with the V866W mutation; it also potently inhibits Gα_q_-mediated PLC-β isozyme activity in cells.

## Results

### Grafting the helix-turn-helix from PLC-β3 into folded proteins

**Figure 1:**
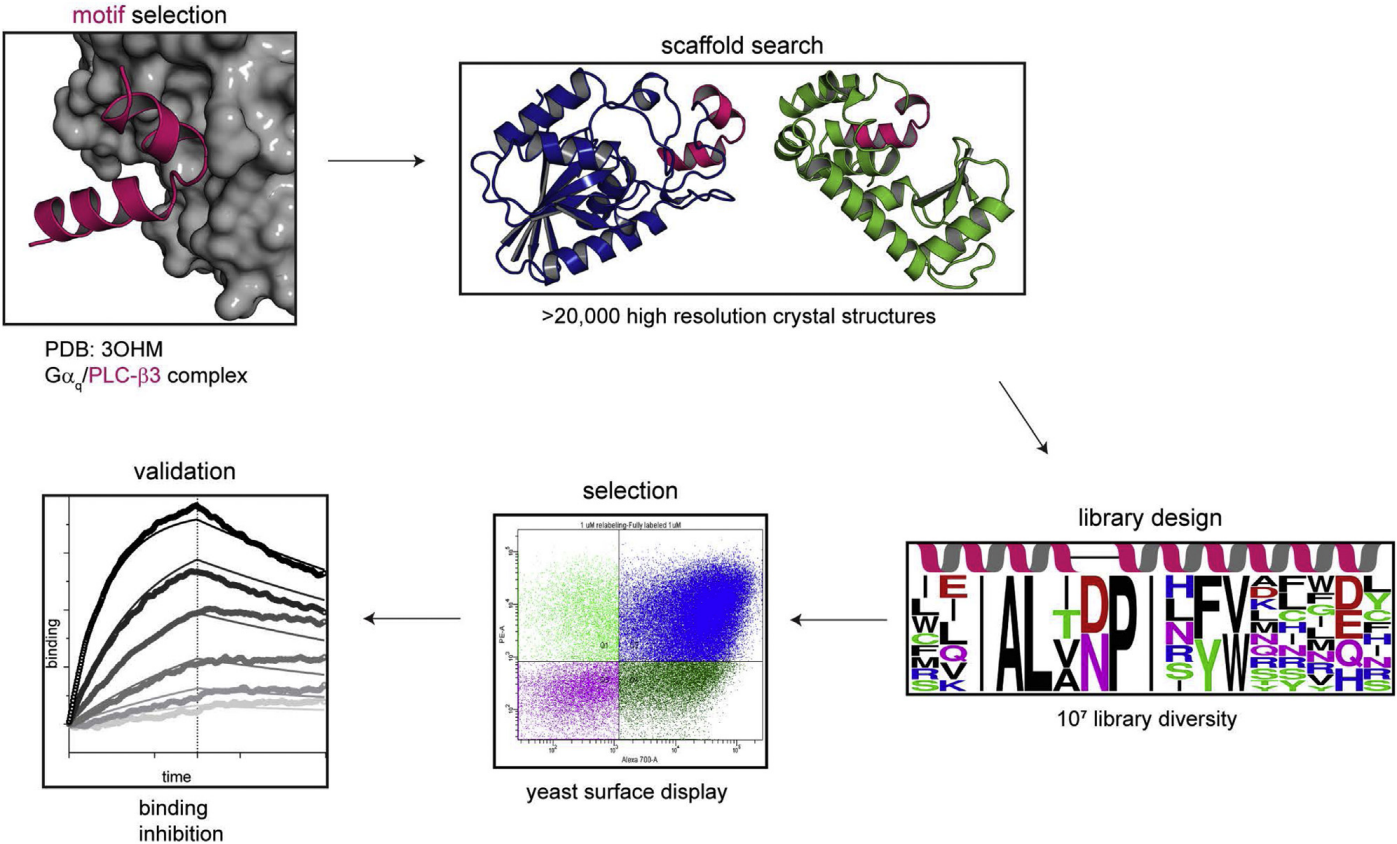
**Workflow for inhibitor design**. *Top left*, the HTH motif (*pink*) from PLC-β3 in complex with activated Gα_q_ (*gray surface*) was the starting point for inhibitor design (PDB ID: 3OHM). The *MotifGraft* protocol in Rosetta searched the protein database (PDB) for proteins (“scaffolds”) with HTH motifs that structurally match the motif from PLC-β3 and can be docked against Gα_q_ without introducing steric clashes. To enhance the affinity of the *MotifGraft* designs for Gα_q_, we used yeast surface display experiments to screen a combinatorial library of mutants. Subsequent experiments included only the top hits.

We used the *MotifGraft* protocol in Rosetta to embed the HTH from PLC-β3 in folded proteins in a conformation favorable for binding Gα_q_ (24) (Fig. 1). In the first stage of the *MotifGraft* protocol, a curated set of more than 26,000 high-resolution X-ray crystal structures of monomeric proteins were searched to find regions of the structure closely aligned in three-dimensional space with the HTH motif. To be considered a match, backbone atoms of a scaffold were required to have less than 1 Å root mean square deviation from the backbone atoms of the HTH when the two structures were aligned. In our first set of simulations, we searched for matches to the entire HTH (residues 852–878 from PDB 3OHM), but no matches were identified. Consequently, we repeated the search using only the region of the HTH critical for binding (residues 855–866) as determined from mutational analysis and examination of the PLC-β3/Gα_q_ crystal structure (7) (Table S1).

In the second stage, the *MotifGraft* protocol superimposes the matched regions of the scaffolds onto the Gα_q_/PLC-β3 structure. Following these superpositions, the protocol checks for clashes between the scaffolds and Gα_q_ to determine if the overall shapes of the scaffolds and matched regions were compatible with binding to Gα_q_. As a result, this step filtered out cases where the HTH aligned to a structural region in the middle of a scaffold, thereby resulting in a poor site to try and introduce a binding surface. Consequently, the first two stages of *MotifGraft* identified 44 potential scaffolds (Table S1).

In the last stage of the *MotifGraft* protocol, the scaffolds were redesigned to accommodate the HTH and, if possible, form additional favorable contacts with Gα_q_. *MotifGraft* performs sequence optimization using fixed backbone rotamer sampling with Rosetta's full atom energy function, which evaluates packing, electrostatic interactions, hydrogen bonding, and desolvation effects (24, 25). During this step, residues in the HTH critical for binding to Gα_q_ (A858, L859, P862, I863) were not allowed to mutate, but residues on the backside of the motif that could potentially form new contacts with the scaffold were allowed to mutate. The outputs from these runs were sorted by the total Rosetta energies of the complexes (normalized by the number of residues) and by the predicted binding energies. Nine proteins (PDB codes: 1EBB, 171L, 1jx6, 1s9h, 1viza, 2x1c, 3chg, 3mx3, and 3rvh) were selected for further optimization with design simulations that allowed more side-chain flexibility (*Fixbb* protocol, see supporting information) and focused on residues at the Gα_q_ interface. We chose the two best scoring models from these simulations, derived from the PDB entries 1EBB and 171L, for experimental studies.

**Figure 2:**
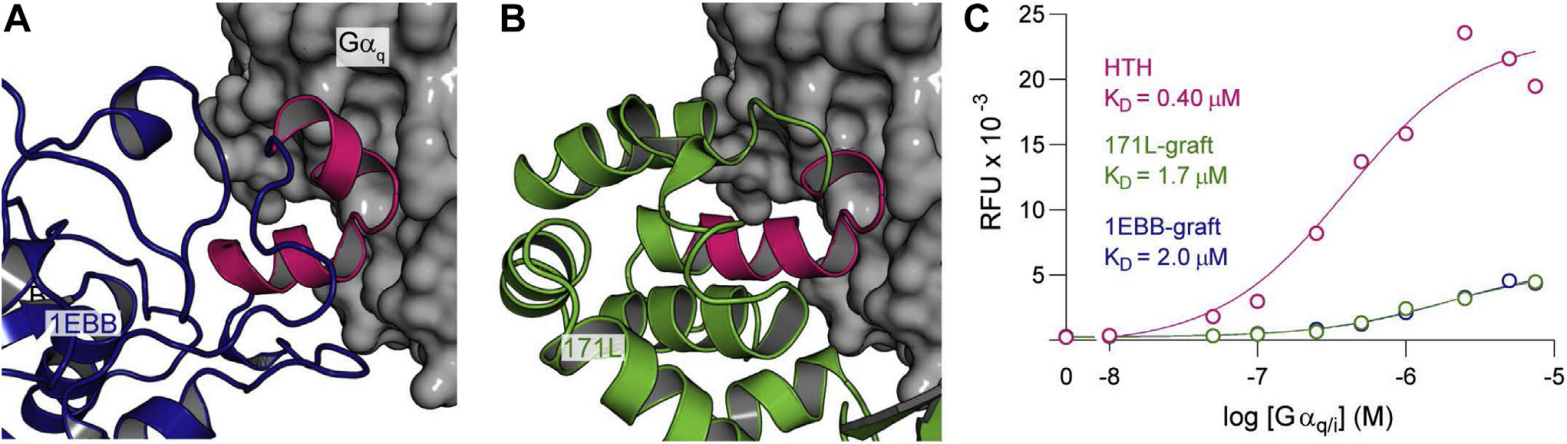
**Validation of Gα**_**q**_**binders identified with *MotifGraft***. *A* and *B*, models of two *MotifGraft* designs (*A*, 1EBB-graft, *B*, 171L-graft) with HTH motifs in *pink* and docked with Gα_q_. 1EBB-graft is derived from a 202-residue hydrolase from *Geobacillus stearothermophilus* (PDB code 1EBB) and 171L-graft is derived from a variant of T4 lysozyme (PDB code 171L). *C*, yeast cell surface titration experiments. Yeast displaying the indicated constructs incubated with increasing concentrations of biotinylated Gα_q/i_ in the presence of AlF4^−^ followed by addition of streptavidin-conjugated to phycoerythrin and subsequent analysis by flow cytometry. The measured relative fluorescence (RFU) fitted to a one-step binding isotherm as a function of Gα_q/i_ concentration, with apparent K_D_ values with 95% confidence intervals: HTH, 0.40 μM (0.23, 0.72); 1EBB-graft, 2.0 μM (1.4, 3.0); 171L-graft, 1.7 μM (1.2, 2.6).

**Figure 3:**
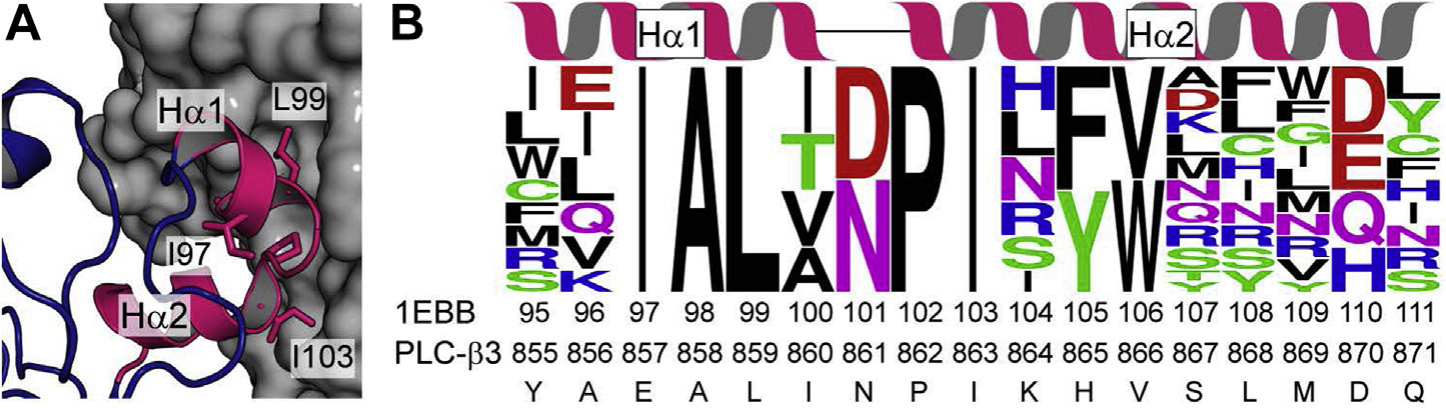
**1EBB-graft directed library**. *A*, model of the HTH region (pink) of 1EBB-graft docked against Gα_q_ (gray surface). The stick-labeled residues were previously identified as necessary for the interaction between PLC-β3 and Gα_q_ or between the HTH and 1EBB scaffold. *B*, a combinatorial library of 1EBB-graft variants incorporated degenerate codons in the HTH region of the protein. The Web Logo in panel B represents the theoretical diversity of the library. The degenerate codons chosen, match amino acid diversity observed in RosettaDesign simulations as well as sequence preferences observed when screening a library of HTH peptide mutants for binding to Gα_q_. Residues (97–99, 102, 103) known to be critical for binding, retained their identities in the library. The sequence of the HTH motif from PLC-β3 is at the bottom of panel B.

1EBB is a 202-residue hydrolase from *Geobacillus stearothermophilus*, and 171L is a variant of T4 lysozyme (26, 27). Superposition of the PLC-β3 HTH with the identified HTH motifs from 1EBB and 171L shows an excellent structural match between these structural elements (Figs. 2, *A* and *B* and 3*A*). For example, a proline that induces a kink between the two helices in the PLC-β3 HTH is found in the equivalent position in both 1EBB (residue 102, residue numbering from the 1EBB crystal structure) and 171L (residue 143). However, outside of the conserved proline, there is low sequence similarity between the PLC-β3 HTH sequence and the WT sequences of structurally aligned regions in 1EBB and 171L: two out of 17 residues are identical between 1EBB and the PLC-β3 HTH, and one out of 17 is identical between 171L and the PLC-β3 HTH (Table 1).

**Table 1:** Sequence alignment of HTH peptides and other engineered proteins

Peptide/Protein	Sequence																										
	852	.	.	.	.	857	.	.	.	.	862	.	.	.	.	867	.	.	.	.	872	.	.	.	.	878	.
WT HTH	H	Q	D	Y	A	E	A	L	I	N	P	I	K	H	V	S	L	M	D	Q	R	A	R	Q	L	A	A
I860A HTH	H	Q	D	Y	A	E	A	L	**A**	N	P	I	K	H	V	S	L	M	D	Q	R	A	R	Q	L	A	A
I86A+V866W HTH (aka “HTH” in all experimental studies)	H	Q	D	Y	A	E	A	L	**A**	N	P	I	K	H	**W**	S	L	M	D	Q	R	A	R	Q	L	A	A
1EBB	.	.	.	D	E	I	R	Q	M	D	P	I	A	F	D	H	F	W	Q	A	.	.	.				
1EBB-graft	.	.	.	**S**	**E**	**I**	A	L	I	**D**	P	I	**N**	**F**	V	**Y**	**F**	**W**	**Q**	**Y**	.	.	.				
1EBB2	.	.	.	**W**	**V**	**I**	A	L	**T**	**D**	P	I	**N**	**F**	**W**	**R**	**Y**	**L**	**E**	**C**	.	.	.				
1EBB25	.	.	.	**W**	**E**	**I**	A	L	**T**	**D**	P	I	**N**	**F**	**W**	**R**	**N**	**N**	D	**L**	.	.	.				
1EBB18	.	.	.	**I**	**E**	**I**	A	L	**V**	**D**	P	I	**R**	**F**	**W**	**T**	**Y**	**R**	D	**H**	.	.	.				
171LWT	.	.	K	S	R	W	Y	N	Q	T	P	N	R	A	K	R	V	I	T	T	.	.	.				
171L-graft	.	.	A	**S**	**K**	**W**	A	L	I	**A**	P	I	**R**	**A**	V	**R**	**V**	**R**	**T**	**T**	.	.	.				
171L-1	.	.	R	**S**	**G**	**W**	A	L	**A**	**T**	P	I	**A**	**A**	**W**	**W**	**F**	**W**	**T**	**R**	.	.	.				
171L-2	.	.	T	**S**	**L**	**W**	A	L	**V**	**N**	P	I	**T**	**Y**	**W**	**L**	**C**	**V**	D	**L**	.	.	.				

Numbering is based on PLC-β3 chain from PDB entry 3OHM. Mutations are bold-faced.

We refer to the two graft designs as 1EBB-graft and 171L-graft (PDB files for the models are provided in supporting information). In both of these designs, *MotifGraft* preserved most of the residues from the scaffold that pack between the HTH motif and the rest of the scaffold. These preserved residues include I97, F105, F108, and W109 from 1EBB and W138, A146, and V149 from 171L. In contrast, residues that point toward solvent in the HTH from 1EBB and 171L were converted to the matching residues in PLC-β3 to induce binding to Gα_q_.

### 1EBB-graft and 171L-graft bind Gα_q/i_ with low micromolar affinity

Initial validation of 1EBB-graft and 171L-graft was performed using binding assays with yeast surface display. Yeast cells expressing 1EBB-graft and 171L-graft as cell surface fusions were incubated with various concentrations of biotinylated Gα_q/i_ activated with AlF_4_^−^, a surrogate for active Gα_q_. Gα_q/i_ is a chimera between Gα_q_ and Gα_i_ that includes all of the PLC-β3 binding elements from Gα_q_, but contains regions of Gα_i_ sequence that allow for robust expression in bacterial cells (sequence in Fig. S2). Our previous binding assays show that peptides derived from the PLC-β3 HTH bind with identical affinities to Gα_q/i_ purified from bacteria and WT Gα_q_ purified from insect cells (20). After incubating the yeast cells with varying amounts of biotinylated Gα_q/i_ for 1-h, fluorescently labeled streptavidin was added, and flow cytometry was used to measure the amount of Gα_q/i_ bound to the surface of the yeast. Data were fit to a one-step binding isotherm and an apparent K_D_ was determined (28). Previous studies have demonstrated the equivalence of binding affinities measured with yeast surface titrations to those obtained using soluble proteins (29). 171L-graft and 1EBB-graft bound to Gα_q/i_ with a K_D_ of 1.7 μM ± 1.5 and 2.0 μM ± 1.7, respectively (Fig. 2*C*). To further check the integrity of our assay, we expressed a variant of the HTH peptide with the mutations I860A and V866W on the surface of yeast. The yeast titration fit to a K_D_ of 400 nM (Fig. 2*C*), which is similar in value to the K_D_ (290 nM) measured for the interaction of a soluble peptide and Gα_q/i_ using fluorescence polarization (Fig. S2). At this stage, we were not surprised that the HTH peptide with the mutations I860A and V866W bound more tightly to Gα_q_ than the grafted designs, as 171L-graft and 1EBB-graft do not contain mutations such as V866W that are at the Gα_q_/PLC-β3 interface and are known to improve binding.

### Affinity matured 1EBB variants show high expression and tight binding to Gα_q/i_

To optimize the affinity of 171L-graft and 1EBB-graft for Gα_q_, we constructed combinatorial libraries that varied the amino acid sequence of the HTH region and selected for tight binders with yeast cell surface display. The library design was based on the sequences of the Rosetta models and 80 sequences identified from an mRNA display screen for HTH peptides that bind tightly to Gα_q_ (personnel communication, Rihe Liu, UNC School of Pharmacy). All mutations were located within the HTH region of both 1EBB and 171L (Figs. 3*B* and S3). During modeling, residues considered “hotspots” (857, 858, 859, 862, and 863 in PLC-β3 numbering) were kept fixed in the library design. The library contained mutations on both sides of the HTH motif, *i.e.*, residues predicted to interact with Gα_q_ as well as residues that pack against the host scaffold (1EBB or 171L). The online tool *SwiftLib* was used to pick degenerate codons that match the desired amino acid profiles at each residue position (30). The theoretical amino acid diversity of each library was >1 × 10^9^. The experimentally obtained diversity of each library was estimated to be on the order of ∼1 × 10^7^. Despite undersampling the library's theoretical diversity, we decided to proceed with selection as we expected that a significant fraction of the library would bind to Gα_q_.

Several rounds of fluorescent-activated cell sorting (FACS) were performed with each directed library. Briefly, each library was subjected to five (171L library) to seven (1EBB library) rounds of FACS while sorting for both the expression of the scaffolds using a c-Myc epitope tag as well as the capacity of the scaffolds to bind varying concentrations of Gα_q/i_ (FACS data for 1EBB library is shown in Fig. S4). In the first rounds of sorting, yeast expressing mutant proteins were labeled with 1 μM biotinylated Gα_q/i_ (activated with AlF4^−^), and sorted cells from this round were labeled with successively lower concentrations of Gα_q/i_ in subsequent rounds to both apply selection pressure for tight binders and eliminate low-affinity binders. The last sort of each library was performed at 4 nM Gα_q/i_. After the final round of sorting with each library, clones were randomly picked, sequenced, and tested for both expression of the scaffolds on the surface of yeast and interaction with Gα_q/i_. Sequencing revealed three unique clones from the 1EBB library (1EBB2, 1EBB18, and 1EBB25) and two unique clones from the 171L library (171L1 and 171L2) (Tables 1 and S2). When examined with yeast display as monoclonal populations, the 1EBB clones showed full-length expression and tight binding to Gα_q/i_. At the same time, the designs based on 171L had relatively poor expression and bound weakly to Gα_q/i_.

### 1EBB25 is folded and binds Gα_q/i_ with nanomolar affinity

**Figure 4:**
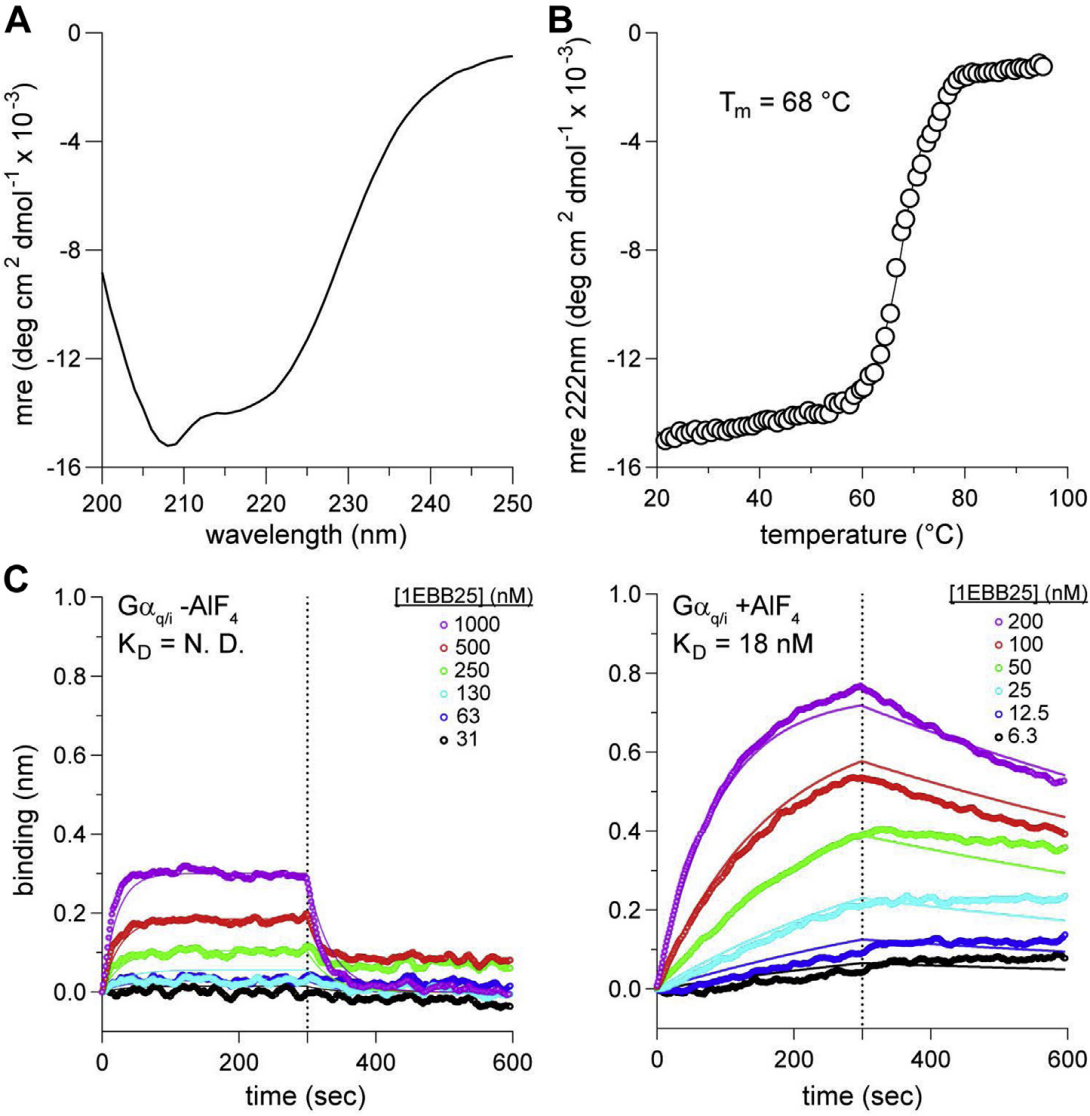
**1EBB25 is stable and binds Gα**_**q/i**_**with high affinity**. *A*, circular dichroism (CD) spectra of purified 1EBB25 indicate that the protein is folded and has significant helical character as expected. *B*, temperature denaturation of 1EBB25 as monitored by CD at 222 nm. The data fitted with the Gibbs–Helmholtz equation, quantified a thermal midpoint for unfolding (T_M_) of 68 °C. *C*, binding analysis of 1EBB25 to Gα_q/i_ by BLI with and without AlF_4_^−^. Biotinylated Gα_q/i_ bound to a streptavidin sensor, and 1EBB25 incubated with the sensor at the indicated concentrations. The equilibrium dissociation constant (K_D_) in the presence of AlF_4_^−^ was measured to be 18 ± 0.22 nM with a *k*_*on*_ and *k*_*off*_ of 5.2 ± 0.1 × 10^4^ M^−1^ s^−1^ and 9.5 ± 0.3 × 10^−4^ s^−1^, respectively.

1EBB25 expressed robustly in *E. coli* with a typical yield after His-tag purification of 6 mg/l. Conversely, 1EBB18 was insoluble. Thus, we proceeded with 1EBB25 as the best candidate for inhibiting Gα_q_. The CD spectrum of 1EBB25 is as expected for the native 1EBB fold, indicating a mixture of α-helices and β-strands (Fig. 4*A*). We quantified the thermal stability of 1EBB25 by heating the protein from 20 °C to 95 °C and recording the change in CD signal (ellipticity) at 222 nm (Fig. 4*B*). 1EBB25 unfolds cooperatively with a melting temperature of 68 °C, indicating that the protein is stable at physiological body temperature. Next, bio-layer interferometry (BLI) experiments were used to quantify the affinity of 1EBB25 for Gα_q/i_. In the presence of AlF_4_^−^, the K_D_ was measured to be 18 ± 1 nM with a *k*_*on*_ and *k*_*off*_ of 5.2 ± 0.1 × 10^4^ M^−1^ s^−1^ and 9.5 ± 0.3 × 10^−4^ s^−1^, respectively (Fig. 4*C*). In contrast, binding experiments without AlF_4_^−^ were not accurate enough for a confident fit: C^2^ > 1.0 and R^2^ < 0.95. This switching behavior demonstrates that 1EBB25 is specific for the active form of Gα_q_. Notably, the binding affinity of 1EBB25 for Gα_q/i_ is 16-fold tighter than our tightest HTH peptide, 110-fold tighter than the affinity of 1EBB-graft for Gα_q/i_, and 50-fold tighter than the affinity of full-length PLC-β3 for Gα_q_ (7).

A multiple sequence alignment of the original affinity-matured HTH peptides, 1EBB-graft, and 1EBB25 sheds light on which mutations identified by the yeast display selection likely contribute to the superior affinity of 1EBB25 (Table 1). For example, V866W increased the affinity of the original HTH peptide for Gα_q/i_ over 14-fold; V866W was also selected during affinity maturation of 1EBB-graft, and this substitution likely behaves similarly in 1EBB25. Modeling of V866W onto the structure of PLC-β3 bound to Gα_q_ suggests that the tryptophan packs against A253, R256, and T257 of Gα_q_. In another interesting example, Y868N and F869N were both selected during the affinity maturation of 1EBB-graft to create 1EBB25, yet neither site points toward Gα_q_—rather both face toward the interior of the 1EBB scaffold. Substitutions at these sites may adjust how the HTH is positioned relative to the rest of the scaffold or how the two helices in the HTH are positioned relative to each other.

**Figure 5:**
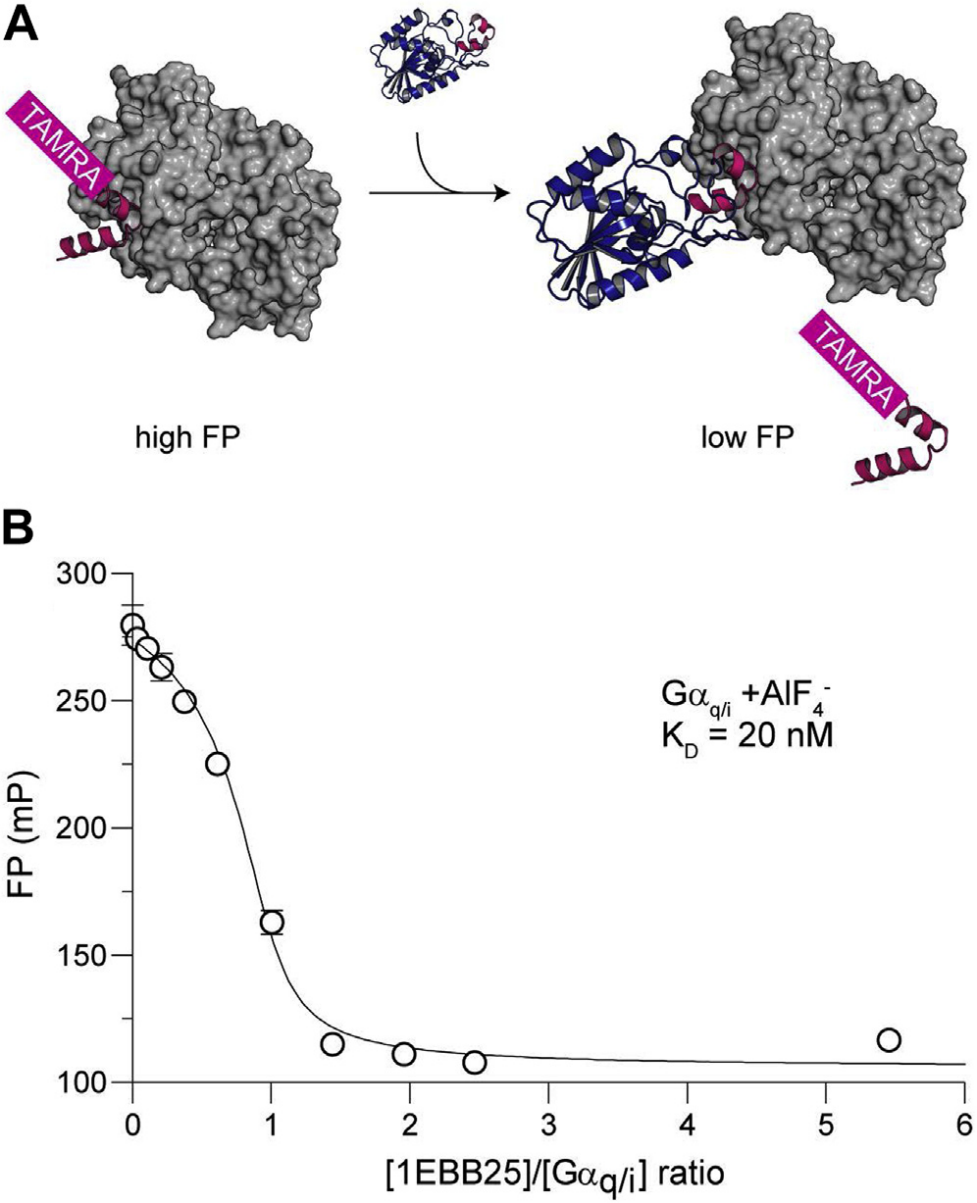
**1EBB25 binds Gα**_**q/i**_**at the desired surface.***A*, schematic representation of the assay. If binding is competitive, then increasing concentrations of 1EBB25 will displace the peptide and lower the measured fluorescence polarization signal. *B*, fluorescence polarization as a function of the concentration of 1EBB25. The Gα_q/i_ and HTH peptide concentrations were 2.75 μM and 400 nM, respectively. The data were fitted with a competitive binding model (see Experimental procedures), which indicated a K_D_ of 20 nM for 1EBB25 with Gα_q/i_, consistent with BLI measurements. Data presented are representative of three independent experiments.

To confirm that 1EBB25 occludes PLC-β isozymes from binding Gα_q_, we performed a competitive binding experiment *in vitro* with 1EBB25 and an HTH peptide derived from PLC-β3 (Fig. 5*A*). Gα_q/i_ was equilibrated with TAMRA-labeled HTH peptide (I860A), and fluorescence polarization was measured as 1EBB25 was titrated into the sample (Fig. 5*B*). A rapid decrease in fluorescence polarization was observed as 1EBB25 displaced the fluorescent peptide from Gα_q/i_. We also performed a direct binding experiment with the TAMRA-labeled HTH peptide (I860A) and Gα_q/I_ and measured a binding affinity of 400 ± 100 nM (20). The efficient displacement of the HTH peptide by 1EBB25 in the competition assay is consistent with the tight affinity we measured between 1EBB25 and Gα_q/i_ in the BLI experiments.

Lastly, we tested if 1EBB25 is specific for Gα_q_ over other G proteins. As the HTH peptide interacts preferentially with Gα_q_ (20), we expected that 1EBB25 would also have high specificity for Gα_q_. We used BLI to test 1EBB25 binding to the other major Gα subunits: Gα_i_, Gα_s_, and Gα_13/i_. The measurements were made in the presence of AlF_4_^−^ to stabilize the G proteins in their active conformation. No measurable affinity was detected for the other Gα subunits (Fig. S5).

Recently a new computational method called AlphaFold was developed for predicting protein structure, and the approach has shown high accuracy in a variety of benchmarks (31). As an independent test of our design model for 1EBB25, we used AlphaFold to predict the structures of 1EBB, 1EBB-graft, and 1EBB25 bound to Gα_q_. The AlphaFold models for 1EBB-graft and 1EBB25 docked with Gα_q_ are nearly identical to our design models while 1EBB is docked in an alternative confirmation that reflects the absence of a favorable binding conformation (Fig. S6). This result further supports that 1EBB25 is binding to the same surface on Gα_q_ that the HTH motif binds to. We also used AlphaFold to predict the structure of 1EBB25 bound to Gα_i_, Gα_s_, Gα_13_, and Gα_11_. Consistent with our binding studies, the models for G_s_, G_13_, and G_i_ did not place 1EBB25 in the position it adopts when binding Gα_q_. However, 1EBB25 is predicted to bind G_11_ in the same docked position as it binds Gα_q_. This result is consistent with the high sequence identity between Gα_q_ and Gα_11_ (>90%).

### 1EBB25 inhibits Gα_q_ signaling

**Figure 6:**
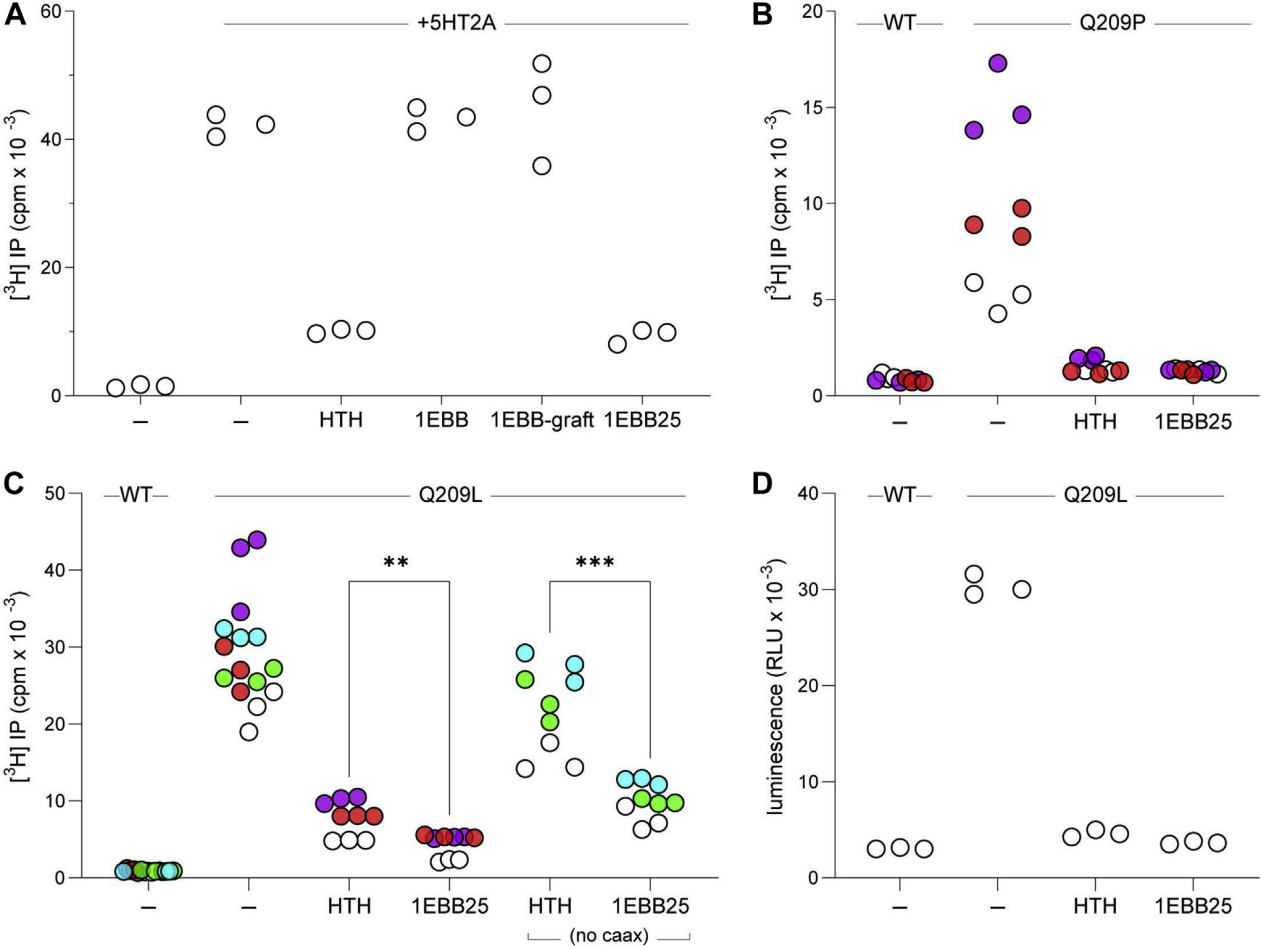
**1EBB25 inhibits Gα**_**q**_**signaling in HEK293 cells**. Unless noted otherwise, all inhibitor constructs were sandwiched between YFP and CFP and contain an N-terminal Myc tag and a C-terminal CAAX box. Within each panel, samples from the same biological replicate are colored the same. *A*, 1EBB25 inhibits WT Gα_q_-mediated activation of PLC-β isozymes. Cells were cotransfected with or without 5HT2A receptor and with or without inhibitors. The activity of PLC-β isozymes was quantified by measuring production of [^3^H] inositol phosphates. *B*, 1EBB25 inhibits Q209P Gα_q_-mediated activation of PLC-β isozymes. Cells were cotransfected with WT or Q209P Gα_q_ and with or without inhibitor. *C*, 1EBB25 inhibits Q209L Gα_q_ with greater efficacy than HTH. The same experiment was performed as in (*B*), but with Q209L Gα_q_. With and without a CAAX box, 1EBB25 showed greater inhibition of Q209L Gα_q_ than the HTH peptide (*D*) 1EBB25 inhibits Q209L Gα_q_-mediated activation of Trio-related RhoGEFs. The experiment was conducted as in (*C*), except the cells were also transfected with a reporter plasmid that expresses luciferase when Trio-related RhoGEFs are activated. Luciferase expression levels were measured by monitoring the luminescence emitted from products of the luciferase reaction.

Encouraged by the tight binding affinity between 1EBB25 and Gα_q/i_, we expressed 1EBB25 in HEK293 cells to test its ability to inhibit Gα_q_-mediated signaling (Fig. 6*A*). Upon stimulation by 2,5-dimethoxy-4-iodoamphetamine (DOI), the 5HT_2A_ receptor activates Gα_q_, which subsequently activates PLC-β isozymes. PLC-β isozymes hydrolyze the phospholipid, phosphatidylinositol 4,5-biphosphate [PtdIns(4,5)P2], into inositol 1,4,5-triphosphate [Ins(1,4,5)P3] and diacylglycerol. Thus, if 1EBB25 can inhibit Gα_q_, the cells will produce less [Ins(1,4,5)P3] upon DOI stimulation. 1EBB25 showed strong inhibition of Gα_q_ signaling, comparable to the HTH peptide (Fig. 6*A*). Consistent with their substantially lower affinity for Gα_q_, neither 1EBBWT nor 1EBB-graft inhibited PLC-β isozymes.

Since Gα_q_ is an oncogene, we sought to measure the inhibition of two oncogenic variants that constitutively activate Gα_q_: Q209P and Q209L. We transfected HEK293 cells with either WT or mutant Gα_q_ and measured the production of [Ins(1,4,5)P3] from cells cotransfected with and without inhibitors. 1EBB25 inhibits both Q209P and Q209L mutants (Fig. 6, *B* and *C*). Moreover, 1EBB25 improves inhibition over the HTH peptide. Given these results, we sought a more stringent test for inhibiting Gα_q_. In a previous study (20), we observed that the HTH peptide does not robustly inhibit WT Gα_q_ without including a CAAX box to localize the protein to the plasma membrane. Thus, we tested the inhibition of Q209L with the HTH peptide and 1EBB25 lacking a CAAX box (Fig. 6*C*). 1EBB25 still exhibits strong inhibition of Q209L even without a CAAX box, while HTH only shows weak inhibition. Interestingly, 1EBB25 only shows a minor improvement in inhibition when expressed with a CAAX box. In all of the cellular experiments described so far, the inhibitors, HTH peptide and 1EBB25, were fused with an N-terminal YFP and a C-terminal CFP. To ensure that the fluorescent proteins were not influencing inhibition, we tested inhibition without the YFP and CFP tags but with the CAAX box (Fig. S7). 1EBB25 still efficaciously inhibited Q209L. Western blots also confirmed that changes in activity were not due to changes in inhibitor expression or G protein expression (Fig. S8).

In a previous study we demonstrated that the HTH peptide can also inhibit Gα_q_-mediated activation of Trio-related RhoGEFs (20). Inhibition is *via* direct competition, as both the HTH and RhoGEFs bind to the same binding cleft on Gα_q_. To test if 1EBB25 can block activation of Trio-related RhoGEFs, we transfected HEK293 cells with plasmids containing genes for Gα_q_ (WT or Q209L), inhibitor (HTH or 1EBB25), and firefly luciferase. The luciferase gene was placed downstream of a serum response element (SRE) promoter, thus making luciferase expression sensitive to the activation of Trio-related RhoGEFs (32). Expression of Gα_q_:Q209L led to robust expression of luciferase in the absence of inhibitors, while only basal levels of luciferase were observed when HTH or 1EBB25 was coexpressed with Gα_q_:Q209L (Fig. 6*D*). These results indicate that 1EBB25 effectively blocks the interaction between activated Gα_q_ and Trio-related RhoGEFs.

## Discussion

In this study, we examined if embedding the HTH peptide in a folded protein could be used to generate a potent inhibitor for Gα_q_. Our engineered protein, 1EBB25, binds to Gα_q/i_ with a K_D_ 18 nM, 16-fold tighter than the HTH peptide variant with the V866W mutation. Embedding a binding motif in a folded protein can potentially boost affinity *via* multiple mechanisms. For example, preordering the motif in the unbound state can reduce how much conformational entropy is lost upon binding, and the additional surface area presented by the scaffold protein can allow the formation of new contacts with the target protein. However, in our models of 1EBB-graft and 1EBB25, the scaffolds do not make additional contacts with Gα_q_, suggesting that the increase in binding affinity we observe is likely derived from stabilizing the HTH in a binding-competent conformation as well as by specific mutations that have been made within the HTH binding motif.

Our approach for improving affinity, *i.e.*, stabilization of the bound conformation in the unbound state, is similar in concept to the use of stapled peptides. Stapled peptides are locked into a unique conformation, typically helical, by introducing covalent bonds between amino acid side chains positioned on the same side of the helix (33). Stapled helices have been developed for various important therapeutic targets such as MDM2 and the estrogen receptor, and they have been observed to bind their targets up to 50-fold tighter than their unstapled analogs (33). These gains in binding affinity are similar in magnitude to what we observed by prestabilizing the HTH using protein scaffolds. When exploring alternative strategies for engineering Gα_q_ inhibitors, we also tested stapled variants of the HTH peptide. Staples were introduced in either the first or second helix of the motif, and binding affinity was measured for Gα_q_. No significant increases in binding affinity were observed (Fig. S9). It is interesting to compare this result with our finding that embedding the HTH in a folded protein can increase affinity. It suggests that correctly positioning the two helices relative to each other is essential for achieving tight binding.

The *MotifGraft* protocol that we used in this study relied on identifying naturally occurring proteins with structural regions that closely align with the HTH. The advantage of this approach is that there is a high likelihood that final engineered proteins will be a stable, folded protein and will adopt the desired structure. A shortcoming of this approach is that one is limited to structures found in the PDB. In our case, we were able to find 44 structures with regions that aligned with the HTH, but less than ten had favorable Rosetta scores once docked onto Gα_q_ and none of these provided an opportunity to design new contacts between the scaffold and the target. An alternative approach to *MotifGraft* is to design a *de novo* protein that incorporates the binding motif. This approach is challenging because it relies more on structure-based modeling, but a few recent successes highlight the usefulness of this approach (34, 35). In addition to providing a mechanism for stabilizing the bound conformation, the new protein can be engineered to make new contacts with the target. One example of this advantage is the recent design of BCL2 inhibitors that bind with high specificity to particular family members (34). In this study, the initial binding motif was a single helix that was incorporated in *de novo* three-helix bundles that make extensive interactions with the target protein *via* the additional helices in the design. It is possible that recent improvements in *de novo* design will allow for a similar approach with the HTH motif (36) and provide a mechanism for simultaneously stabilizing the bound conformation and introducing new contacts at the Gα_q_ interface.

Since 1EBB25 is genetically encoded, it has potential utility in a variety of applications. For example, we are currently testing if genes encoding for Gα_q_ inhibitors can be delivered to Gα_q_-driven uveal melanoma cells with adeno-associated virus in order to inhibit tumor growth. Another valuable feature of 1EBB25 is that it binds specifically to the active form of Gα_q_ and therefore may help create biosensors that precisely monitor the activation state of Gα_q_ in cells or can be used to purify active Gα_q_ from cell lysate for proteomic studies. In general, our work demonstrates that embedding binding motifs in folded proteins is a valuable approach for engineering tight binders and should apply to many peptide–protein binding interactions.

## Experimental procedures

### Rosetta modeling

The Rosetta application *MotifGraft* (24) was used to search for naturally occurring proteins (“scaffolds”) that contain regions of structure with backbone atoms that align closely in three-dimensional space with the backbone atoms from the HTH motif of PLC-β3 bound to Gα_q_. The input for the simulation included: (1) the complete HTH motif (27 amino acids), or several truncated forms, from the PLC-β3 chain of the PDB entry 3OHM (Table S1), (2) the Gα_q_ chain from the same PDB entry as the partner structure, and (3) a curated set of high-resolution crystal structures of 26,775 monomeric proteins (gift from William Schief's Laboratory, The Scripps Institute, USA). *MotifGraft* outputs models of the putative scaffolds docked onto Gα_q_. *MotifGraft* docked the scaffolds by rotating and translating the scaffold so that the residues that match the HTH motif were structurally superimposed onto the HTH motif in the Gα_q_/PLC-β3 crystal structure. However, the scaffold now has to accommodate the incoming HTH residues and new potential contacts with Gα_q_. Therefore, the sequence of the scaffold was optimized with three rounds of fixed-backbone rotamer-based sequence design in Rosetta (37). Only scaffold residues in or near the HTH motif were allowed to vary during these simulations, and residues known to be critical for binding were kept fixed. A list of fixed residues in the form of a “resfile” and command line options are detailed in the supplemental methods. For each round of *Fixbb* design, models were chosen based on the total score generated by each simulation and visual inspection.

### Binding titration of top models with yeast display

The yeast display vector pETCON (38) and the yeast strain EBY100 (gifts from David Baker's laboratory at the University of Washington) were used to express the designed proteins on the surface of yeast. Genes for the designed proteins were incorporated into the yeast display vector by cotransforming yeast with linearized vector and insert DNA that contained the gene sequence. The inserts were ordered (gblocks from IDT DNA) or prepared as dsDNA with 50 bp regions at the 5′ and 3′ ends that are identical in sequence to the 3′ and 5′ ends of the linearized vector, respectively. The sequence homology at the ends of the insert and linearized vector leads to homologous recombination in yeast and the creation of circularized plasmids incorporating the gene of interest. The DNA sequences of all templates and primers are provided in Tables S2 and S3, respectively.

A gene of the HTH was amplified by PCR from a previously prepared pTriEx4 plasmid containing the HTH sequence. In cases where the designed genes were not ordered with the flanking sequences needed for homologous recombination with the yeast display vector, the flanking sequences were added by PCR. A 50 μl PCR reaction was composed of Q5 1× reaction buffer (NEB), 200 μM dNTP mix, 1 μl template DNA (∼100 ng), 1 U Q5 high-fidelity DNA polymerase (NEB), and the respective primers (MWG Operon) at 0.5 μM. The PCR mixture was subjected to thermocycling as follows: denaturation at 98 °C for 30 s, followed by 30 cycles of denaturation, annealing and extension at 98 °C (10 s), 64 °C (30 s), and 72° (30 s), respectively, followed by a final extension step of 5 min at 72 °C. The pETCON vector was digested with NheI, BamHI, and SalI restriction endonucleases (Fermentas). The vector (125 ng) and insert (725 ng) DNA were cotransformed *via* electroporation (Biorad) in electrocompetent yeast EBY100 cells (28). After an hour of growth in YPD medium (10 g/l yeast extract, 20 g/l peptone, 20 g/l dextrose) at 30 °C and 250 rpm, cells were pelleted and resuspended in 10 ml SDCAA medium (20 g/l dextrose, 5 g/l casamino acids, 6.7 g/l yeast nitrogen base, 5.40 g/l Na_2_HPO_4_, 7.45 g/l NaH_2_PO_4_). Subsequently, 100 μl of cells was plated on an SDCAA plate (20 g/l dextrose, 5 g/l Casamino acids, 6.7 g/l yeast nitrogen base, 182 g/l sorbitol, 5.40 g/l Na_2_HPO_4_, 7.45 g/l NaH_2_PO_4_, 15 g/l agar) for growth of single colonies in a 30 °C incubator for 2 days. DNA sequencing of single colonies (by zymoprep kit, Zymoresearch followed by sequencing by Genewiz) was used to verify incorporation of the gene of interest. All sequencing from yeast display vector (pETCON) was performed using the forward primer: 5′-GTTCCAGACTACGCTCTGCAGG-3′.

To induce protein expression, cells were inoculated in SGCAA medium (20 g/l galactose, 5 g/l Casamino acids, 6.7 g/l yeast nitrogen base, 5.40 g/l Na_2_HPO_4_, 7.45 g/l NaH_2_PO_4_) at a starting OD_600_ of 1 and grown for ∼20 h in a shaker at 20 °C, 250 rpm. Protein expression was analyzed by flow cytometry (BD FACS Canto) after labeling induced yeast cells with anti-c-myc antibody followed by goat anti-chicken antibody conjugated to Alexa 633. All antibodies and labeling reagents for the yeast display work were purchased from Thermo Fisher Scientific unless otherwise stated. To test if the designed proteins bind to Gα_q/i_, yeast surface titrations were performed. For all labeling, the following buffer was made fresh (Gα_q_ buffer): 10 mM HEPES (pH 8.0), 150 mM NaCl, 10 mM MgCl_2_, 30 μM AlCl_3_, 10 mM NaF, and 0.1% BSA. SGCAA-induced yeast cells were washed with the labeling buffer followed by primary labeling with AlF_4_^−^ activated, biotinylated Gα_q/i_ at concentrations ranging from 1 nM to 7.5 μM. After 1 h of incubation on ice with gentle rotation, cells were washed, and secondary labeling with 4 μg/ml diluted streptavidin conjugated to phycoerythrin (SA-PE) was performed for 10 min on ice. Cells were washed and analyzed in a BD FACS Canto flow cytometer (BD Biosciences), and the mean fluorescence intensity was recorded in the PE channel for 50,000 events. The K_D_ of interaction was determined using the following relationship:

$$F=\;\frac{c\left[L_{0}\right]}{K_{D}+L_{0}}$$

where *F* is the observed mean fluorescence intensity, [*L*]_0_ is Gα_q/i_ concentration, and *c* is a constant. The constants *c* and *K*_*D*_ were fitted by a nonlinear least squares regression in Microsoft Excel (Solver add on), and the error between the fitted curve and observed data was estimated in terms of 68% confidence interval.

### Directed library design and construction for affinity maturation

To affinity mature the Rosetta-based designs, combinatorial libraries were created with degenerate codons at residue positions at or near the Gα_q_ binding site. The degenerate codon used at each residue position was determined using a multistep process. At first, the amino acid profiles were constructed for each residue position (Figs. 3*B* and S3) by combining results from the Rosetta design simulations and peptide-display experiments that were performed with variants of the HTH peptide (personnel communication Rihe Liu, UNC). A degenerate codon optimization tool that we have created, called *SwiftLib*, (http://rosettadesign.med.unc.edu/SwiftLib/) was then used to identify degenerate codons that would best match the desired amino acid profile at each position while maintaining a library size well suited for yeast display (30). Input and output obtained from *SwiftLib* for the 1EBB library design are given in Tables S4 and S5, respectively.

To accommodate all the degenerate codons, eight oligos were ordered as building blocks for library generation (Table S6). Insert DNA was prepared as two cassettes, B12 and B2 with 39 bp of homology between them (the first half of the insert was B12 and the second half B2). B12 was generated by first PCR amplifying the eight library oligos generating cassette B11 followed by a second PCR with B11 as a template to generate the final B12 cassette. Each 50 μl PCR (eight reactions) to synthesize the B11 cassette was composed of 1× Q5 reaction buffer (NEB), 200 μM dNTP mix, 0.5 μM each of the 1EBBLibFwd1 and 1EBBLibInsert1Rev primers (IDT DNA), mix of all eight oligos at 1 μM each and 1 U high-fidelity Q5 DNA polymerase (NEB). The reaction mixture was denatured at 98 °C for 30 s followed by 30 cycles of denaturation at 98 °C for 10 s, annealing at 62 °C for 30 s, extension at 72 °C for 30 s, and a final extension at 72 °C for 5 min in an Eppendorf Mastercycler Pro machine. Next, the B12 cassette was synthesized by PCR-amplifying B11. Each 50 μl PCR to synthesize B12 cassette was composed of 1× Q5 reaction buffer (NEB), 200 μM dNTP mix, 0.5 μM each of the 1EBBLibFwd2 and 1EBBLibInsert1Rev primers (IDT DNA), B11 at 3.5 ng/μl, and 1 U high-fidelity Q5 DNA polymerase (NEB). Cycling condition to synthesize B12 was as described for B11. Cassette B2 was synthesized by PCR-amplifying a gblock (IDT DNA) using the composition and cycling condition described above with 1 μl of 10 nM template gblock and 1EBBLibInsert2Fwd and 1EBB-graft reverse primer in a 50 μl PCR. The 1EBB yeast display library was generated using the protocols described above with ∼60 μg of B12 and B2 each and 20 μg digested pETCON vector by electroporation of 20 × 0.2 cm gap cuvettes (USA scientific). Yeast colonies grown on serially diluted SDCAA plates gave an estimated library diversity of ∼1.1 × 10^7^. The directed library for the 171L design was constructed using a similar protocol. Briefly, *SwiftLib* was used to first design desired oligonucleotides (Tables S7–S9). The 171L gene was split into two cassettes L1 and L2 for transformation into yeast. The L1 fragment, which has no mutations, was generated by PCR amplifying the L1 gblock (IDT DNA) using the primers listed with the PCR composition and cycling as described before for the B2 gblock (Tables S2 and S3). The L2 cassette harbors the desired mutations and was synthesized by PCR amplifying the 171L-Lib oligos (Table S9) as described above with the appropriate primers (Table S3). L1 and L2 were designed to have a 42 base pair region of homology between them. Library transformation was performed as described for the 1EBB library. Colony count on serially diluted SDCAA plates gave an estimated library diversity of ∼1 × 10^7^.

### FACS and individual clone analysis

Expression of the yeast library was induced by switching yeast cell culture medium from SDCAA to SGCAA (20 g/l galactose, 2 g/l dextrose, 5 g/l Casamino acids, 6.7 g/l yeast nitrogen base, 5.40 g/l Na_2_HPO_4_, 7.45 g/l NaH_2_PO_4_) for 20–24 h at 20 °C and 250 rpm. Each library was subjected to several rounds of FACS. For each round of sorting, induced yeast cells from SGCAA culture were first washed with freshly made Gα_q_ buffer and labeled with varying concentrations of biotinylated Gα_q/i_ (starting at 1 μM in the first sort down to 4 nM in the last sort) and 4 μg/ml chicken anti-c-Myc IgY (Thermo Fisher scientific) for an hour of incubation at 4 °C with gentle rotation. After washing off unbound reagents, secondary labeling was performed with 4 μg/ml goat anti-chicken Alexa-633 conjugate (ThermoFisher scientific) and 4 μg/ml streptavidin-phycoerythrin (SA-PE) to achieve immunofluorescent detection. All sorts were performed in a Beckman Coulter MoFlo XDP flow cytometer located at the UNC Flow Cytometry core facility. Cells washed after secondary labeling were pelleted and kept on ice until they were analyzed in the flow cytometer. All appropriate controls were performed as described previously (39). Five and seven rounds of sorting were performed for the 171L and 1EBB libraries, respectively. The population isolated from the last round of sorting was plated on an SDCAA/agar plate followed by culture into SDCAA media. Twenty-nine clones from the 1EBB lineage and 11 clones from the 171L lineage were tested for c-myc expression and binding to Gα_q/i_ (at 4 nM) followed by sequencing (Eurofin Genomics).

### Cloning for bacterial and mammalian expression

For expression in *E. coli*, 1EBB mutants isolated from yeast display were cloned into pCDB24 vector (gift from The Baker Lab, University of Washington) as a C-terminal fusion to Sumo using the Gibson Assembly mix (NEB). For each gene, a 50 μl PCR was composed of 1× Q5 reaction buffer (NEB), 200 μM dNTP mix, 0.5 μM forward and reverse primers (as in Table S3, synthesized by Eurofin Genomics), 100 ng template DNA, and 1 U high-fidelity Q5 DNA polymerase (NEB). The reaction mixture was denatured at 98 °C for 30 s followed by 30 cycles of denaturation at 98 °C for 10 s, annealing at 64 °C for 30 s, extension at 72 °C for 30 s, and a final extension at 72 °C for 5 min pCDB24 vector was digested with XhoI (Fermentas) enabling cloning of 1EBB gene(s) following an N-terminal Sumo protein and UlpI cleavage site. A typical Gibson Assembly reaction was composed of 0.025 pmol vector and 0.25 pmol insert pooled into a 20 μl reaction with a final 1× Gibson Assembly mix (G.A.). G.A. reaction was then incubated at 50 °C for 1 h followed by chemical transformation into DH5α cells. Accuracy of cloning was verified by DNA sequencing (Eurofin Genomics). Sequence-verified plasmids were transformed in BL21(DE3)pLysS cells to enable *E. coli* expression. For mammalian expression, all 1EBB genes were codon-optimized (IDT DNA) and cloned in pTriEx4 vector as a fusion between YFP (N-terminus) and CFP (C-terminus) followed by Rac1 CAAX box (CAAX box sequence: PPPVKKRKRKCAIL) (Template and primers in Tables S2 and S3). For ease of expression in *E. coli*, a chimeric form of Gα_q_ containing a small portion of Gα_i1_ (termed Gα_q/i_) was used. This chimeric form retained the PLCβ-3 binding site (Fig. S1). Gα_q/i_ (with or without an avitag) was cloned in pET15b vector that has an N-terminus 6XHis tag followed by TEV cleavage site. To enable *in vivo* biotinylation, an avitag sequence (GLNDIFEAQKIEWHE) was fused to the C-terminus of Gα_q/i_. To enable *E. coli* expression, pET15b harboring Gα_q/i_ gene was transformed in BL21 (DE3) cells and pET15b harboring Gα_q/i_-avitag was transformed in T7 Express cells (NEB) together with pBirA vector, which contains the biotin ligase BirA (Avidity Technologies, pBirAcm).

### Recombinant expression and purification of proteins

1EBB25 was expressed in BL21 pLysS cells. First, 50 ml of LB media (with 100 μg/ml ampicillin) was inoculated with a single colony of BL21 cells and grown overnight at 37 °C, 200 rpm. The overnight culture was diluted 1:100 in 1.5 L LB media supplemented with ampicillin at 37 °C, 200 rpm. Expression of proteins was induced by adding IPTG (1 mM) to the culture at an OD_600_ of 0.6 to 0.8. Cells were harvested after 2.5 h of induction at 37 °C, 200 rpm. Cell pellets were frozen at −80 °C for an hour (or until ready to purify), followed by resuspension in 30 ml lysis buffer/1.5 L expression [lysis buffer: 50 mM Na_2_HPO_4_, pH 8.0, 300 mM NaCl, 20 mM imidazole, 2 mM 2-mercaptoethanol]. Cells were homogenized by sonication, followed by lysate recovery by centrifugation. Supernatant from the sonicated (Fisher) cell pellets was purified with 5 ml Ni-NTA resin (Qiagen) in a 25 ml gravity column (Biorad). Sumo fusions of 1EBB25 from Ni-NTA purification were simultaneously dialyzed and cleaved with UlpI [dialysis buffer: 25 mM HEPES, pH8.0, 150 mM NaCl]. Cleaved proteins were further purified with a S75 gel filtration column (GE) in a GE AKTA FPLC system.

G proteins were also expressed and purified with metal affinity chromatography. Each liter of TB media was inoculated with sufficient overnight culture to reach an OD600 of 0.1. The cultures were incubated at 37 °C, 225 rpm until the OD_600_ reached 1.8, expression was then induced with the addition of 0.2 mM IPTG and incubated overnight at 20 °C, 225 rpm. Cell pellets were harvested by centrifugation for 30 min at 4000 rpm in a Beckman J6-MI swinging-bucket centrifuge and weighed. Pellets were stored at −80 °C until processed. All steps were performed at 4 °C. Frozen pellets were suspended in lysis buffer (20 mM Tris pH 8.0, 300 mM NaCl, 10% glycerol, 5 mM imidazole, 10 mM MgCl_2_, 30 μM AlCl_3_, 10 mM NaF, 15 mM GDP, 2 mM 2-mercaptoethanol) at 5 ml of buffer per gram of cells. Lysis was performed using an EmulsiFlex-C5; three passes at 10,000 PSI ensured all cells were lysed. The lysate was centrifuged for 60 min at 64.2 K RCF and the supernatant was collected. Ni-NTA agarose resin (Qiagen, 30230) was added to the supernatant and incubated on a shaker at ∼ 60 rpm for 1 h. The bound resin was loaded into a gravity flow column and washed with four column volumes (CV) of lysis buffer and four CV wash buffer [Lysis buffer with 50 mM imidazole]. For biotinylation, avi-tagged G proteins were washed on column with ten CV of 10 mM Tris pH 7.4 and 10 mM MgCl_2_. Following wash, resin was then incubated in one CV of 10 mM Tris pH 7.4, 10 mM MgCl_2_, 1× BiomixB (Avidity), and 0.25 mg/ml BirA ligase for 1 h at room temperature. G proteins were eluted with four CV of elution buffer [Lysis buffer containing 300 mM Imidazole]. The eluate was concentrated to ∼6 ml using Vivaspin 30 kDa 20 ml concentrators (Sartorious, VS2022) and purified over a 120 ml Sephacryl S-200 HR (GE Healthcare, 17-0584-01) column. Monomeric G proteins eluted at ∼43 kDa while soluble aggregates eluted in the void volume. The monomeric G proteins were pooled, concentration measured, and treated overnight with TEV at 20 μg TEV/mg G protein on ice. The digested G protein was run over a 1 ml HisTrap HP (GE Healthcare 17-5247-01) and the flow-through was collected. The G proteins were concentrated to ∼6 ml again and purified over a 120 ml Superdex 75 column (GE Healthcare 17104401) to remove any trace contaminants (size exclusion buffer: 20 mM Tris pH 8.0, 150 mM NaCl, 5% glycerol, 10 mM MgCl_2_, 30 μM AlCl_3_, 10 mM NaF, 15 mM GDP, 2 mM DTT). The final product was pooled, concentrated with 10 kDa filters to 10 to 20 mg/ml, and flash-frozen with liquid nitrogen for storage at −80 °C.

### Circular dichroism (CD)

All CD experiments were performed with a JASCO J-815 CD spectrometer. Data were collected with protein concentrations between 30 and 100 μM in a 1 mm path-length cuvette at 20 °C with a scanning speed of 50 nm/min between 190 to 260 nm wavelength using 4 s data integration time, 2 nm bandwidth, and 1 nm pitch. Ellipticity data were normalized to mean residue ellipticity, θ [deg·cm^2^·dmol^−1^] using the equation: $\theta =\frac{100\;\times \;mdeg}{c\;\times \;\left(N-1\right)\times \;l}$ where *c*, *N*, and *l* refer to concentration (mM), number of amino acid residues, and optical path length (cm). Thermal denaturation was observed by monitoring the CD signal at 222 nm from 20 °C to 95 °C. Data were fitted to the Gibbs–Helmholtz equation by a nonlinear least squares regression in Microsoft Excel.

### Bio-layer interferometry (BLI)

BLI experiments were performed by initially equilibrating ForteBio streptavidin sensors (18-5019) in a 96 well plate in 250 μl of BLI buffer: 20 mM HEPES pH 7.4, 150 mM NaCl, 10 mM MgCl_2_, 30 μM AlCl_3_, and 1× ForteBio kinetics buffer (18-1092). Sensors were transferred to a separate plate containing three columns of 250 μl wells: (1) BLI buffer, (2) biotinylated G protein (0.025 mg/ml), and (3) variable concentrations of 1EBB25. The steps for the sensors were the following: custom step for 1 min in (1), loading step for 5 min in (2), baseline step for 5 min in (1), association step for 5 min in (3), and finally dissociation step for 5 min in (1). Experiments were done with and without 10 mM NaF. The temperature of the instrument was set to 30 °C.

The sensorgrams were analyzed using ForteBio Data Analysis software. The sensorgrams were first aligned at the *y* axis of the association step. An inter-step correction was applied to the association step. The sensorgrams were processed using Savitzky-Golay filtering. Global curve fitting was performed on both association and dissociation steps.

### Fluorescence polarization

For competitive FP assays, TAMRA-labeled HTH-pep (0.4 μM) and Gα_q/i_ (2.75 μM) were first equilibrated in buffer containing 20 mM HEPES pH 7.4, 150 mM NaCl, 10 mM MgCl_2_, 30 μM AlCl_3_, and 10 mM NaF. Titration of 1EBB mutants was then performed at the concentrations indicated in Figure 6. All FP experiments were conducted in a Jobin Yvon Horiba FluoroMax3 fluorescence spectrometer. The equilibrium dissociation constant for the designed proteins was determined by fitting the FP data with a numerical model of competitive binding. Details can be found in the supporting information.

### AlphaFold prediction of 1EBB/G protein complexes

AlphaFold predictions were made using the ColabFold framework (40). In short, each complex was predicted by enforcing a 1:1 stoichiometry. A separate MSA was generated for each protein using the mmseq2 web server (41). Five models were predicted, and the prediction with the lowest overall RMSD to the 1EBB-graft/Gα_q_ Rosetta model is shown (Fig. S6). Amber relax and templates were not used during the prediction.

### Cell culture inhibition assay and immunoblotting

Cell-based assays for measuring the accumulation of [^3^H]-inositol phosphates were performed as described previously (7, 20). Briefly, HEK293A cells in 12-well plate were transfected with 100 ng 5HT2A receptor together with 100 ng 1EBB or HTH-pep constructs using Continuum (Gemini Bio-products). Empty pcDNA was added for total of 300 ng of DNA per well. Twenty-four hours posttransfection, cells were labeled with 1 μCi of [^3^H]*myo*-inositol in serum-free, inositol-free DMEM for 16 h followed by a 1 h stimulation with 2 μM DOI in the presence of 10 mM LiCl. Cells were lysed with formic acid followed by neutralization with ammonium hydroxide, and the lysate was subsequently applied to Dowex column. Accumulation of [^3^H]-inositol phosphates was measured by liquid scintillation counting. Expression of 1EBB and HTH-pep constructs was probed with a monoclonal antibody against GFP (Clonetech, catalog # 632380) or Myc (Invitrogen 46-0603). G proteins were probed with a monoclonal antibody against HA (BioLegend 901501). As a loading control, lysates were also probed with an antibody against β-actin (Clone-AC15, Sigma-Aldrich). Detection of both primary antibodies was achieved by using a goat anti-mouse IgG-HRP antibody followed by the addition of BioRad Clarity ECL substrate. Blots were imaged by ChemiDoc Imaging Systems (Biorad).

Inhibition assay for oncogenic Gα_q_ was modified slightly. Instead of 100 ng of 5HT2A receptor, 10 ng of oncogenic Gα_q_ was transfected. Inhibitor constructs were decreased from 100 ng to 20 ng. Final DNA amount remained as 300 ng per well. No DOI was required as the Gα_q_ mutants are constitutively active.

Inhibition assay using SRE-luciferase was performed using a Pierce Firefly Luciferase Glow Assay Kit (Catalog# 16176). The assay was performed per manufacturer's instructions. In short, ∼10,000 HEK293A cells/well were seeded onto a 96-well plate. After ∼24 h, cells were transfected with 1 ng of Gα_q_ (WT or Q209L), 2 ng of inhibitor, 10 ng of SRE-luciferase, and enough empty pcDNA to reach 100 ng total DNA. After ∼24 h of expression, media were replaced with serum-free DMEM for ∼18 h. Finally, cells were lysed and luciferase activity was quantified on a clariostar plate reader.

## Data availability

The Rosetta molecular modeling program is available from the RosettaCommons consortium (https://www.rosettacommons.org/software).

## Supporting information

This article contains supporting information.

## Conflict of interest

The authors declare that they have no conflicts of interest with the contents of this article.
