# Supplementary material for: Designer proteins that competitively inhibit Gαq by targeting its effector site
Source: J Biol Chem. 2021 Oct 27;297(6):101348. doi: 10.1016/j.jbc.2021.101348 (PMC8633581; doi:10.1016/j.jbc.2021.101348)
Supplement: Supporting information 1 [file mmc1.pdf]

Supporting Information

Designer proteins that competitively inhibit Gα_q by targeting its effector site

Mahmud Hussain[1], Matthew C. Cummins[2], Stuart Endo-Streeter[2], John Sondek[1, 2, 3], Brian Kuhlman[1, 3*]

[1] Department of Biochemistry and Biophysics, University of North Carolina, Chapel Hill, North Carolina, USA, [2]Department of Pharmacology, University of North Carolina, Chapel Hill, North Carolina, USA, [3]Lineberger Comprehensive Cancer Center, University of North Carolina, Chapel Hill, North Carolina, USA

List of material

Modeling by Rosetta: code and options....S-2

MotifGraft....S-2

Fixbb....S-3

Details of numerical modeling of competitive fluorescence polarization data....S-4

Table S1: Summary of motifs used as input to *MotifGraft* and output PDB's obtained from the simulations....S-5

Table S2: Sequence of template DNA....S-6

Table S3: List of Primers....S-8

Table S4: Input to *SwiftLib* to design 1ebb directed library....S-9

Table S5: *SwiftLib* output with degenerate codons for 1ebb directed library design....S-10

Table S6: Oligonucleotides used to generate 1EBB library (synthesized by Eurofin Genomics) S-11

Table S7: Representative input to *SwiftLib* to design 171l directed library....S-12

Table S8: *SwiftLib* output with degenerate codons for 171l directed library design....S-12

Table S9: Oligonucleotides used to generate 171l library (synthesized by Eurofin Genomics) S-13

Figure S1: 21-mer HTH with the V866W mutation specifically binds the active form of G$\alpha_{q/i}$.. S-15

Figure S2: Sequence alignment of Gα_q, Gα_{i1} and G$\alpha_{q/i}$....S-16

Figure S3: Design of 171l directed library....S-17

Figure S4: FACS analysis of 1ebb yeast library....S-18

Figure S5: BLI experiment testing binding of 1EBB25 to other major Gα protein: Gα i, Gα s, and Gα 13/i....S-19

Figure S6: AlphaFold predictions recapitulate Rosetta model....S-20

Figure S7: Inhibition of Gα_q_Q209L by HTH and 1EBB25 without YFP and CFP....S-21

Figure S8: Western blots of co-transfection experiments....S-22

Figure S9 Competitive fluorescence polarization assay was performed similar to Fig. 6........S-23

Modeling by Rosetta: code and options

MotifGraft

An example of minimal xml code that was used to run *MotifGraft* simulations is shown below:

```
"<MotifGraft name="motif_grafting"
context_structure="./3OHMContextStructure.pdb"
motif_structure="./motif_1052to1062_3OHM.pdb"
RMSD_tolerance="1.0" NC_points_RMSD_tolerance="1.0"
clash_score_cutoff="5" clash_test_residue="GLY"
hotspots="3:4:7" combinatory_fragment_size_delta="0:0"
max_fragment_replacement_size_delta="0:0"
full_motif_bb_alignment="1"
allow_independent_alignment_per_fragment="0"
graft_only_hotspots_by_replacement="0"
only_allow_if_N_point_match_aa_identity="0"
only_allow_if_C_point_match_aa_identity="0"
revert_graft_to_native_sequence="1"
allow_repeat_same_graft_output="0"/>"
```

As outlined above, some of the user-definable options chosen were: (1) An RMSD tolerance of 1 °A upon superimposition of the input motif with the scaffold backbone, (2) N and C-terminus alignment tolerance of the motif with the scaffold backbone was also set to 1 °A, (3) Clash score residue was set to 'Gly' for all clash-tests, (4) The maximum number of atomic clashes to be tolerated was set to 5, (5) Upon successful grafting, clash test was turned off for mutating the 'hot spot' residues as defined for a given input motif which is numbered by individual motif pdb's. For instance hotspots "3:4:7" refers to the 3rd, 4th and 7th residue of the input motif in the above example, (6) The full backbone alignment was set to "1" meaning the size of the fragment to be replaced has to be exactly the same size of the Motif fragment, and (7) Grafted motif was reverted back to the native scaffold sequence except for the 'hot spot' residues. Simulations were performed at the UNC super computer cluster with a representative command for a production run as following:

```
"bsub -q day -n 175 -J 3OHMfullMotifgraft -o log.%J -B -N -a
mvapich mpirun
~/GIT/Rosetta/main/source/bin/rosetta_scripts.mpi.linuxgccreleas
e -database ~/GIT/Rosetta/main/database/ -overwrite -
out:file:renumber_pdb -ex1 -ex2 -
mpi_work_partition_job_distributor -mpi_tracer_to_file proc -
```

```
parser:protocol -ignore_zero_occupancy false -out:output -
parser:protocol epigraft.xml -l Allmonomers -nstruct 20"
```

Fixbb

All simulations used 'talaris2013' as the energy function. A typical run for a *Fixbb* simulation on a Macbook computer (Apple) was as following with the associated texts from the 'options' and 'resfile' for a model generated from the PDB 1ebb:

Command line

```
"~/GIT/Rosetta/main/source/bin/fixbb.macosgccrelease
@flags_fullatom_dun10 -resfile resfile -database
~/GIT/Rosetta/main/database/ > log.txt"
```

'Options' file text:

```
"-s 1ebba_0001.pdb
-database /nas02/home/m/h/mhussain/GIT/Rosetta/main/database/
-dun10
-out:file:o 1ebba_0001_.pdb
-nstruct 10

-ex1
-ex2
-use_input_sc
-extrachi_cutoff 8
-resfile resfile

-minimize_sidechains
-run::min_type dfpmin_armijo
-score:weights talaris2013.wts

-mpi_work_partition_job_distributor
-mpi_tracer_to_file proc"
```

'resfile' text:

```
"NATAA
start

#re-designing to force some of the Motif sidechains as obtained
from MotifGraft

413 B NOTAA C
414 B NOTAA C
415 B NOTAA C
```

```
416 B PIKAA L
417 B PIKAA I
418 B NOTAA C
419 B NOTAA C
420 B PIKAA I
421 B NOTAA C
422 B NOTAA C
423 B PIKAA WV
424 B NOTAA C"
```

Notably, only residues that were within the grafted motif were allowed to mutate with the following restrictions: (1) No mutations to Cysteine, (2) Some 'hotspot' residues that were known to contribute to binding, and (3) Both Valine and Tryptophan were allowed in residue Val866 (numbering as in PDB 3ohm but shown as residue 423 above in the resfile.). Trp866 was chosen based on previously identified V866W mutation from screening an HTH-peptide library against Gα_q. For each round of *Fixbb* design, models were chosen based on total score generated by each simulation and visual inspection.

Details of numerical modeling of competitive fluorescence polarization data

In this model, the competitive binding experiment is represented as:

$$[Gq] + [TAMRApep] \underset{k_{-1}}{\overset{k_1}{\rightleftharpoons}} [Gq\ TAMRApep]$$

$$[Gq] + [competitor] \underset{k_{-2}}{\overset{k_2}{\rightleftharpoons}} [Gq\ competitor]$$

The change in concentration of each species as a function of time can be written as:

$$\Delta[Gq] = k_{-1}[Gq\ TAMRApep] - k_1[Gq][TAMRApep] + k_{-2}[Gq\ competitor] - k_2[Gq][competitor]$$

$$\Delta[TAMRApep] = k_{-1}[Gq\ TAMRApep] - k_1[Gq][TAMRApep]$$

$$\Delta[competitor] = k_{-2}[Gq\ competitor] - k_2[Gq][competitor]$$

$$\Delta[Gq\ TAMRApep] = -k_{-1}[Gq\ TAMRApep] + k_1[Gq][TAMRApep]$$

$$\Delta[Gq\ competitor] = -k_{-2}[Gq\ competitor] + k_2[Gq][competitor]$$

Since the rate constants can be rewritten in terms of the dissociation constant, an arbitrary rate constant was used to rewrite the above equations:

$$K_D = \frac{k_{-1}}{k_1}$$

$$K_D * k_1 = k_{-1}$$

$$arbitrarily\ set\ k_1 = 1$$

$$\Delta[Gq] = K_{D\ pep}[Gq\ TAMRApep] - [Gq][TAMRApep] + K_{D\ comp}[Gq\ competitor] - [Gq][competitor]$$

$$\Delta[TAMRApep] = K_{D\ pep}[Gq\ TAMRApep] - [Gq][TAMRApep]$$

$$\Delta[competitor] = K_{D\ comp}[Gq\ competitor] - [Gq][competitor]$$

$$\Delta[Gq\ TAMRApep] = -K_{D\ pep}[Gq\ TAMRApep] + [Gq][TAMRApep]$$

$$\Delta[Gq\ competitor] = -K_{D\ comp}[Gq\ competitor] + [Gq][competitor]$$

Where $K_{D\ pep}$ or $K_{D\ comp}$ are the dissociation constants between Gq and TAMRApep or competitor respectively. Since $K_{D\ pep}$ and monomer concentrations are all known, these equations can be used to simulate the steady state concentrations of all species for a given $K_{D\ comp}$. The simulated FP signal is estimated as

$$simulated\ FP = fraction\ of\ bound\ TAMRApep * FP_{max} = \frac{[Gq\ TAMRApep]}{total\ [TAMRApep]} * FP_{max}$$

Different values of $K_{D\ comp}$ are simulated to identify the best fit to the experimentally obtained FP curve.

Supporting Tables

Table S1: Summary of motifs used as input to *MotifGraft* and output PDB's obtained from the simulations

Motif sequence	Residue Numbering by PDB 3ohm	Number of matching scaffolds	PDB ID of output structures*
HQDYAEALINPIKHVSLMDQRARQLAA	852 to 878	0	N/A
YAEALINPIKHVS	855 to 867	26	1ebb, 1h2e, 1h2f, 1ri5, 1viz, 2vd7, 2ybk, 3chg, 3dxt, 3g87, 3i3c, 3njy, 3pdq, 3rcq, 3rgi, 3rvh, 3sbm
YAEALINPIKHV	855 to 866	44	1bja, 1ebb, 1fmt, 1gxy, 1gxz, 1gy0, 1h2e, 1h2f, 1jx6, 1s9h, 1viz, 2b4m, 2b7w, 2b7x, 2vd7, 2xml, 2ybk, 3chg, 3dxt, 3g87, 3i3c, 3njy, 3pdq, 3pl7, 3q0i, 3rcq, 3rgi, 3rio, 3rvh, 3sbm, 3tc8, 3tyk, 1711
AEALINPIKHVS	854 to 867	25	1ebb, 1h2e, 1h2f, 1r0w, 1s9h, 2vd7, 2ybk, 3i3c, 3mx3, 3njy, 3pdq, 3rcq, 3rio, 3rvh, 3sgh, 3tc8
AEALINPIKHV	854 to 866	45	1fmt, 1gxy, 1gxz, 1gy0, 1jx6, 1q7s, 1r0w, 1s9h, 2ae0, 2gae, 2pic, 2pjj,

			2vd7, 2x1c, 2x1e, 2ybk, 3dxt, 3i3c, 3mx3, 3njy, 3pdq, 3q0i, 3rcq, 3rio, 3rvh, 3sgh, 3tc8, 3tyk

* For some PDB's, multiple matching output was obtained.

Table S2: Sequence of template DNA

Clone	DNA sequence
HTH-pep	5′-CATCAGGATTATGCGGAAGCGCTGGCGAACCCGATTAAACATTGGA<br>GCCTGATGGATCAGCGCGCGCGCCAGCTGGCGGCG-3′
1EBB-graft	5′-CTAGTGGTGGAGGAGGCTCTGGTGGAGGCGGTAGCGGAGGCGGAGGG<br>TCG GCTAGCGCGACCACCCTCTACCTTACTCGTCACGGCGAAACTAAGT<br>GGAATGTGGAACGCCGTATGCAGGGTTGGCAGGATTCCCCACTTACAGAA<br>AAAGGACGCCAGGATGCAATGCGCCTGGGCAAGCGTCTGGAAGCCGTCGA<br>GCTGGCCGCCATTTATACTTCGACTAGCGGCCGCGCTTTAGAAACTGCAG<br>AGATTGTGCGCGGTGGCCGTTTGATTCCGATCTACCAGGATGAACGCCTG<br>CGCGAAATTCACCTTGGCGATTGGGAGGGAAAAACACATTCGGAAATTGC<br>CTTGATCGATCCGATCAATTTCGTCTATTTTTGGCAGTATCCGCATTTGT<br>ATGCGCCGCAGCGCGGGGAACGGTTTGCGGATGTGCAGCAGCGCGCGTTA<br>GAAGCTGTCCAAAGCATTGTTGATCGCCATGAAGGAGAGACGGTACTGAT<br>CGTGACGCATGGAGTTGTACTGAAGACCCTGATGGCAGCTTTTAAAGACA<br>CCCCGTTAGATCATCTGTGGTCGCCCCCGTATATGTACGGGACCAGCGTC<br>ACGATCATCGAAGTGGATGGTGGTACATTTCATGTCGCGGTGGAAGGTGA<br>TGTCAGTCATATTGAGGGATCCGAACAAAAGCTTATTTCTGAAGAGGACT<br>TGTAATAGCTCGAGATCTGATA-3′
1711-graft	5′-GGCTAGCATGAATATTTTTGAGATGCTGCGCATTGACCATGGACTGC<br>GTCTTAAGATTTACAAGTCATCCGAAGGATACTACGCGATTGGTATTGGG<br>CATCTTCTGACCAAAAGCCCAAGCCTGAATGCGGCAAAAAGTGCATTAGA<br>TAAAGCCATCGGACGGAACACCAATGGTGTCATTACTAAAGATGAAGCAG<br>AAAAGCTTTTTAACCAGGATGTCGACGCTGCCGTGCGTGGTATTCTGCGC<br>AACGCTAAGCTGAAACCAGTGTACGATTCCCTGGATGCGGTTCGTCGTGC<br>GGCTCTGATTAATATGGTATTTCAGATGGGTGAGACTGGCGTCGCCGGCT<br>TCACTAATATCCTGCGGATGTTGCAGCAAAAACGTTGGGATGAAGCCGCC<br>AAGAATATGGCTGCTAGCAAATGGGCCTTGATCGCGCCCATTCGCGCGGT<br>GCGCGTGCGCACAACCTTACAGACGGGCACCTGGGATGCTTACAAAGGAT<br>CCG-3′
1EBB25 (isolated from yeast library)	5′-GCGACCACCCTCTACCTTACTCGTCACGGCGAAACTAAGTGGAATGT<br>GGAACGCCGTATGCAGGGTTGGCAGGATTCCCCACTTACAGAAAAAGGAC<br>GCCAGGATGCAATGCGCCTGGGCAAGCGTCTGGAAGCCGTCGAGCTGGCC<br>GCCATTTATACTTCGACTAGCGGCCGCGCTTTAGAAACTGCAGAGATTGT<br>GCGCGGTGGCCGTTTGATTCCGATCTACCAGGATGAACGCCTGCGCGAAA<br>TTCACCTTGGCGATTGGGAGGGGAAGACGCACTGGGAAATTGCCTTGACA<br>GACCCGATCAACTTCTGGCGGAACAACGACCTCCCGCATTTGTATGCGCC<br>GCAGCGCGGGGAACGGTTTGCGGATGTGCAGCAGCGCGCGTTAGAAGCTG<br>TCCAAAGCATTGTTGATCGCCATGAAGGAGAGACGGTACTGATCGTGACG<br>CATGGAGTTGTACTGAAGACCCTGATGGCAGCTTTTAAAGACACCCCGTT<br>AGATCATCTGTGGTCGCCCCCGTATATGTACGGGACCAGCGTCACGATCA<br>TCGAAGTGGATGGTGGTACATTTCATGTCGCGGTGGAAGGTGATGTCAGT<br>CATATTGAG-3′
1EBB18 (isolated from yeast library)	5′-GCGACCACCCTCTACCTTACTCGTCACGGCGAAACTAAGTGGAATGT<br>GGAACGCCGTATGCAGGGTTGGCAGGATTCCCCACTTACAGAAAAAGGAC<br>GCCAGGATGCAATGCGCCTGGGCAAGCGTCTGGAAGCCGTCGAGCTGGCC<br>GCCATTTATACTTCGACTAGCGGCCGCGCTTTAGAAACTGCAGAGATTGT<br>GCGCGGTGGCCGTTTGATTCCGATCTACCAGGATGAACGCCTGCGCGAAA<br>TTCACCTTGGCGATTGGGAGGGGAAGACGCACATCGAAATTGCCTTGGTA

	GACCCGATCCGCTTCTGGACCTACAGGGACCACCCGCATTTGTATGCGCC<br>GCAGCGCGGGGAACGGTTTGCGGATGTGCAGCAGCGCGCGTTAGAAGCTG<br>TCCAAAGCATTGTTGATCGCCATGAAGGAGAGACGGTACTGATCGTGACG<br>CANGGAGTTGTACTGAAGACCCTGATGGCAGCTTTTAAAGACACCCCGTT<br>AGATCATCTGTGGTCGCCCCCGTATATGTACGGGACCAGCGTCACGATCA<br>TCGAAGTGGATGGTGGTACATTTCATGTCGCGGTGGAAGGTGATGTCAGT<br>CATATTGAG-3′
1EBB2 (isolated from yeast library)	5′-CGCGACCACCCTCTACCTTACTCGTCACGGCGAAACTAAGTGGAATG<br>TGGAACGCCGTATGCAGGGTTGGCAGGATTCCCCACTTACAGAAAAAGGA<br>CGCCAGGATGCAATGCGCCTGGGCAAGCGTCTGGAAGCCGTCGAGCTGGC<br>CGCCATTTATACTTCGACTAGCGGCCGCGCTTTAGAAACTGCAGAGATTG<br>TGCGCGGTGGCCGTTTGATTCCGATCTACCAGGATGAACGCCTGCGCGAA<br>ATTCACCTTGGCGATTGGGAGGGGAAGACGCACTGGGTAATTGCCTTGAC<br>AGACCCGATCAACTTCTGGCGGTACTTGGAATGCCCGCATTTGTATGCGC<br>CGCAGCGCGGGGAACGGTTTGCGGATGTGCAGCAGCGCGCGTTAGAAGCT<br>GTCCAAAGCATTGTTGATCGCCATGAAGGAGAGACGGTACTGATCGTGAC<br>GCATGGAGTTGTACTGAAGACCCTGATGGCAGCTTTTAAAGACACCCCGT<br>TAGATCATCTGTGGTCGCCCCCGTATATGTACGGGACCAGCGTCACGATC<br>ATCGAAGTGGATGGTGGTACATTTCATGTCGCGGTGGAAGGTGATGTCAG<br>TCATATTGAG-3′
171l-1 (isolated from yeast library)	5′ATGAATATTTTTGAGATGCTGCGCATTGACCATGGACTGCGTCTTAAG<br>ATTTACAAGTCATCCGAAGGATACTACGCGATTGGTATTGGGCATCTTCT<br>GACCAAAAGCCCAAGCCTGAATGCGGCAAAAAGTGCATTAGATAAAGCCA<br>TCGGACGGAACACCAATGGTGTCATTACTAAAGATGAAGCAGAAAAGCTT<br>TTTAACCAGGATGTCGACGCTGCCGTGCGTGGTATTCTGCGCAACGCTAA<br>GCTGAAACCAGTGTACGATTCCCTGGATGCGGTTCGTCGTGCGGCTCTGA<br>TTAATATGGTATTTCAGATGGGTGAGACTGGCGTCGCCGGCTTCACTAAT<br>ATCCTGCGGATGTTGCAGCAAAAACGTTGGGATGAAGCGGCCAAGAACAT<br>GGCCAGGTCGGGATGGGCCTTGGCAACCCCCATTGCGGCATGGTGGTTCT<br>GGACACGCCTTCAGACGGGCACCTGGGATGCTTACAAA-3′
171l-2 (isolated from yeast library)	ATGAATATTTTTGAGATGCTGCGCATTGACCATGGACTGCGTCTTAAGAT<br>TTACAAGTCATCCGAAGGATACTACGCGATTGGTATTGGGCATCTTCTGA<br>CCAAAAGCCCAAGCCTGAATGCGGCAAAAAGTGCATTAGATAAAGCCATC<br>GGACGGAACACCAATGGTGTCATTACTAAAGATGAAGCAGAAAAGCTTTT<br>TAACCAGGATGTCGACGCTGCCGTGCGTGGTATTCTGCGCAACGCTAAGC<br>TGAAACCAGTGTACGATTCCCTGGATGCGGTTCGTCGTGCGGCTCTGATT<br>AATATGGTATTTCAGATGGGTGAGACTGGCGTCGCCGGCTTCACTAATAT<br>CCTGCGGATGTTGCAGCAAAAACGTTGGGATGAAGCGGCCAAGAACATGG<br>CCACGTCGCTATGGGCCTTGGTAAACCCCATTACGTACTGGTTGTGCGTG<br>GACCTGCTTCAGACGGGCACCTGGGATGCTTACAAA
1EBB-graft (codon optimized for transfection into HEK293 cells)	5′ATGTATCCTTATGACGTACCAGACTACGCTGCAACTACATTGTATTTG<br>ACGCGACACGGCGAAACCAAATGGAATGTCGAGCGCCGGATGCAAGGCTG<br>GCAGGACAGTCCACTCACGGAAAAAGGTCGCCAGGATGCGATGCGACTGG<br>GCAAACGATTGGAAGCCGTCGAACTGGCGGCGATATATACATCCACATCT<br>GGGAGAGCCCTTGAAACAGCGGAAATCGTTAGGGGAGGGAGACTGATCCC<br>TATTTACCAGGACGAGAGACTTAGAGAAATACACCTGGGTGACTGGGAGG<br>GCAAGACGCATTCCGAGATTGCGCTTATCGATCCCATTAACTTTGTTTAC<br>TTCTGGCAGTACCCCCACCTTTACGCGCCTCAACGAGGGGAGAGATTTGC<br>CGATGTACAGCAAAGAGCCTTGGAGGCTGTGCAAAGTATTGTGGATAGGC<br>ATGAGGGAGAGACTGTGTTGATCGTCACACATGGTGTGGTCCTTAAGACC<br>CTCATGGCGGCATTTAAGGACACACCTCTTGACCACCTCTGGAGCCCACC<br>ATACATGTATGGGACATCTGTGACCATCATTGAGGTAGACGGCGGCACCT<br>TTCACGTCGCCGTTGAAGGCGACGTATCTCACATCGAA-3′
1EBB25 (codon optimized for transfection into HEK293 cells)	5′-ATGTACCCCTACGATGTTCCAGATTACGCAGCGACTACACTCTATTT<br>GACAAGACACGGTGAGACCAAGTGGAACGTCGAACGCCGAATGCAGGGGT<br>GGCAAGACAGTCCGCTGACAGAGAAAGGTCGCCAAGATGCGATGCGACTG<br>GGCAAGCGGCTGGAGGCCGTAGAACTGGCGGCTATCTACACCAGTACATC<br>CGGGCGAGCCCTTGAAACAGCAGAGATCGTTCGGGGTGGCCGGTTGATAC<br>CTATATATCAAGACGAGAGGCTCAGAGAAATCCATCTGGGTGATTGGGAG<br>GGGAAGACGCATTGGGAGATAGCGCTGACGGATCCTATAAACTTCTGGAG<br>GAATAACGACCTTCCACATTTGTATGCGCCCCAGCGAGGCGAGCGATTTG<br>CAGATGTGCAACAGAGAGCTTTGGAAGCTGTGCAAAGTATCGTTGATAGA<br>CATGAGGGCGAGACAGTATTGATCGTAACTCACGGGGTAGTGCTTAAAAC<br>TCTGATGGCTGCCTTCAAGGACACACCGCTCGATCATCTTTGGTCCCCAC<br>CATACATGTATGGAACAAGTGTCACAATCATTGAAGTGGATGGAGGCACG<br>TTCCATGTGGCTGTGGAAGGTGACGTATCTCACATCGAG-3′
1EBB18 (codon optimized for transfection into HEK293 cells)	5′-ATGTATCCGTATGATGTGCCTGACTATGCCGCTACCACACTGTACCT<br>GACGCGCCACGGTGAGACGAAGTGGAATGTCGAACGCCGGATGCAAGGAT<br>GGCAAGATAGCCCGCTGACTGAGAAGGGCAGGCAGGACGCAATGAGGCTG<br>GGCAAGCGCCTCGAAGCAGTTGAGCTGGCCGCTATCTATACTTCCACCAG

	CGGGCGAGCTCTGGAGACTGCGGAAATAGTACGCGGAGGGCGACTTATTC<br>CGATTTACCAGGATGAACGACTGCGAGAAATCCACCTGGGGGATTGGGAA<br>GGCAAGACTCATATAGAAATTGCATTGGTAGATCCGATACGGTTCTGGAC<br>TTATAGAGACCACCCCCATCTGTACGCCCCTCAACGCGGAGAGAGGTTTG<br>CTGATGTACAGCAACGAGCACTCGAGGCTGTACAGTCCATCGTCGATCGC<br>CACGAAGGAGAAACGGTACTGATTGTTACTCACGGTGTGGTGTTGAAGAC<br>ATTGATGGCTGCTTTCAAGGACACTCCACTTGACCATCTGTGGAGTCCCC<br>CGTATATGTACGGTACATCAGTAACTATAATCGAGGTTGACGGCGGCACG<br>TTTCACGTTGCTGTCGAGGGGGATGTATCTCACATCGAA-3′
1EBB WT (codon optimized for transfection into HEK293 cells)	5′-ATGTACCCCTATGATGTTCCCGATTATGCCGCTACTACTTTGTACTT<br>GACCCGCCATGGAGAGACTAAGTGGAACGTGGAGAGGCGCATGCAGGGGT<br>GGCAGGATAGCCCCCTTACTGAAAAGGGAAGACAGGATGCGATGAGACTG<br>GGGAAGCGCTTGGAGGCCGTAGAGCTGGCCGCGATTTATACAAGCACGAG<br>TGGGAGGGCGCTGGAAACCGCCGAGATAGTGCGCGGTGGTCGGCTTATCC<br>CCATATACCAGGATGAAAGACTTCGAGAAATACATCTCGGGGACTGGGAG<br>GGCAAGACCCACGACGAGATCCGCCAAATGGACCCCATCGCTTTCGACCA<br>CTTTTGGCAAGCACCCCATCTGTACGCCCCACAGCGGGGGGAGAGATTTT<br>GTGATGTGCAACAGAGAGCACTCGAAGCCGTCCAGTCAATAGTTGACCGG<br>CACGAGGGGGAGACGGTATTGATAGTGACCCATGGTGTGGTGCTGAAGAC<br>TTTGATGGCTGCTTTCAAAGATACACCACTGGACCACCTGTGGAGTCCAC<br>CCTACATGTACGGCACTAGCGTCACGATCATCGAGGTCGATGGCGGAACC<br>TTCCACGTCGCCGTGGAGGGAGATGTGTCCCATATCGAA-3′
B2 gblock	5′-CCGCATTTGTATGCGCCGCAGCGCGGGGAACGGTTTGCGGATGTGCA<br>GCAGCGCGCGTTAGAAGCTGTCCAAAGCATTGTTGATCGCCATGAAGGAG<br>AGACGGTACTGATCGTGACGCATGGAGTTGTACTGAAGACCCTGATGGCA<br>GCTTTTAAAGACACCCCGTTAGATCATCTGTGGTCGCCCCCGTATATGTA<br>CGGGACCAGCGTCACGATCATCGAAGTGGATGGTGGTACATTTCATGTCG<br>CGGTGGAAGGTGATGTCAGTCATATTGAGGGATCCGAACAAAAGCTTATT<br>TCTGAAGAGGACTTGTAATAGCTCGAGATCTGATA-3′
L1 gblock	5′-GGCTAGCATGAATATTTTTGAGATGCTGCGCATTGACCATGGACTG<br>CGTCTTAAGATTTACAAGTCATCCGAAGGATACTACGCGATTGGTATTGG<br>GCATCTTCTGACCAAAAGCCCAAGCCTGAATGCGGCAAAAAGTGCATTAG<br>ATAAAGCCATCGGACGGAACACCAATGGTGTCATTACTAAAGATGAAGCA<br>GAAAAGCTTTTTAACCAGGATGTCGACGCTGCCGTGCGTGGTATTCTGCG<br>CAACGCTAAGCTGAAACCAGTGTACGATTCCCTGGATGCGG-3′

Table S3: List of Primers

**Primer**	**Sequence**
HTH-pep Forward primer (for yeast display cloning)	5′-CTAGTGGTGGAGGAGGCTCTGGTGGAGGCGGTAG<br>CGGAGGCGGAGGGTCGGCTAGCCATCAGGATTATGCG<br>GAAGCG-3′
HTH-pep Reverse primer (for yeast display cloning)	5′-TATCAGATCTCGAGCTATTACAAGTCCTCTTCAG<br>AAATAAGCTTTTGTTCGGATCCCGCCGCCAGCTGGCG<br>CG-3′
1ebb-graft Forward primer (for yeast display cloning)	5′-CTAGTGGTGGAGGAGGCTCT-3′
1ebb-graft Reverse primer (for yeast display cloning)	5′-TATCAGATCTCGAGCTATTACAA-3′

171l-graft Forward primer (for yeast display cloning of 171l-graft and insert 1)	5′-CTAGTGGTGGAGGAGGCTCTGGTGGAGGCGGTAG CGGAGGCGGAGGGTCGGCTAGCATGAATATTTTTGAGA-3′
171l-graft Reverse primer (for yeast display cloning)	5′-TATCAGATCTCGAGCTATTACAAGTCCTCTTCAGAAA TAAGCTTTTGTTCGGATCCTTTGTAAGCATCCC-3′
1ebb_Lib_Fwd1	5′-T GCGCCTGGGCAAGCGTCTGGAAGCCGTCGAGCTGGCCGCCA TTTATACTTCGACTAGCGGCCGCGCTTTAGAAACTGCAGAGATTGT GCGCGGTGGCCGTTTGATTCCGATCTACCAGGATGAACGCCTGCGC GAAATTCACCTTGGCGATTGGGAGGGGAAGACGCAC-3′
1ebb_Lib_Insert1_Rev	5′- CGCAAACCG TTCCCCGCGC TGCGGCGCAT ACAA ATGCGG-3′
1ebb_Lib_Fwd2	5′-CTAGTGGTGGAGGAGGCTCTGGTGGAGGCGGTAGCGG AGGCGGAGGGTCGGCTAGCGCGACCACCCTCTACCTT ACTCGTCACGGCGAAACTAAGTGGAATGTGGAACGC CGTATGCAGGGTTGGCAGGATTCCCCACTTACAGAA AAAGGACGCCAGGATGCAATGCGCCTGGGCAAGCGTC-3′
1ebb_Lib_Insert2_Fwd	5′-CCGCATTTGT ATGCGCCGCA-3′
1ebb_pCDB24_Fwd	5'-CCGTGAACAGATTGGCGGCGCGACCACCC TCTACCT TAC-3'
1ebb_pCDB24_Rev	5'-CTTTGTTAGCAGCCGGATCCTCATTACTCAATATGAC TGACATCACCTT-3'
1ebb_JS1666_Fwd	5'-TACAATCAAAGGAGATATACCATGGAGCAGAAGCTGATCT CCGAGGAGGACCTGGGATCTGGATCTGGATCTGCTACTACTTT GTACTTGACCC-3'
1ebb_JS1666_Rev	5'-GGTGATGGTGATGGTGGTGCTCGAGTTAAAGGATGGCACATTT TCTCTTCCTCTTCTTCACGGGAGGCGGTTCGATATGGGACACATCT CC-3'
171l_Insert1_Lib_Rev	5′- GTACGATTCC CTGGATGCGG TTCGTCGTGC GGCTCTGATT AATATGGTAT TTCAGATGG -3′
171l_Lib_Insert2_Fwd	5′- CGG TTCGTCGTGC GGCTCTGATT AATATGGTAT TTCAGA TGGG TGAGACTGGC GTCGCCGGCT TCACTAATAT CCTGCGGAT G TTGCAGCAAA AACGTTGGGA TGAAGCG GCC AAG AAC ATG GCC-3′
171l_Lib_Insert2_Rev	5′- TATCAGATCTCGAGCTATTACAAGTCCTCTTCAGAAATAAGCTT TTGTTCGGATCCTTTGTAAGCATCCCAGGTGCCCGTCTGAAG-3′
171L_pCDB24_Fwd	5'-CCGTGAACAGATTGGCGGC ATGAATATTT TTGAGATGCT GCG-3'
171L_pCDB24_Rev	5'-CTTTGTTAGCAGCCGGATCCTCATTATTTGTAAGCATCCCA GGTGCC-3'

Table S4: Input to *SwiftLib* to design 1ebb directed library.

Top row indicates residue number as per 1ebb and left-most column indicates amino acid. Only mutated positions are shown in the table. Entries in each cell indicate the frequency of amino acid desired in that position. Additionally, to favor Rosetta predicted mutations, 50% weightage was placed for residues present in 1ebb-graft. For example, at position 95, no Serine was obtained from HTH-pep library sequencing but Serine was predicted by Rosetta at that position (numbering as in 1ebb scaffold). Subsequently, frequency of Serine was set to 40 (50% of the 80 sequences from HTH-pep library). As an optional input, up to 2 degenerate codons were allowed for each position. To penalize STOP codons, frequency was set to -10. Empty cells represent a frequency of ‘0’.

	95	96	97	100	101	104	105	106	107	108	109	110	111
Ala		2	2	3		6			9	3			1
Cys							1			1			
Asp	8		3		22	3			1	1		68	1

Glu		41	6	6		2			1	1		12	1
Phe	6		1	1		2	37		3	46	8		3
Gly													
His	4					2	2		2	5	1		7
Ile	2	12	40	52		1			1	2	16		3
Lys			4			7			11	1	4		4
Leu	7	28	11	5		16			6	22	5		16
Met	9	6	4	7		2			3	4	6		8
Asn	3		1		49	40			7	4	1		2
Pro			2										1
Gln		6	1	2		1			6	1		40	5
Arg	3	4	8			12			17	10	6		15
Ser	40	2	2		9	14			8	4			6
Thr		2	1	18		1				1	1		1
Val	2	14		24				40		1	12		3
Trp	32	1	16			5	11	40		7	51		
Tyr	4	1	8	1		2	29		42	5	9		41
Stop	-10	-10	-10	-10	-10	-10	-10	-10	-10	-10	-10	-10	-10

Table S5: *SwiftLib* output with degenerate codons for 1ebb directed library design.
Numbering as in 1ebb scaffold.

Pos	Stretch	Codon	Present	Absent	Error	# Codons	# AA	%desired
95	1	NAC, WKS*	C(0) D(8) F(6) H(4) I(2) L(7) M(9) N(3) R(3) S(40) W(32) Y(4)	V(2)	2	12	12	92%
96	1	VWA	E(41) I(12) K(0) L(28) Q(6) V(14)	A(2) M(6) R(4) S(2) T(2) W(1) Y(1)	18	6	6	83%
97	1	WKS	C(0) F(1) I(40) L(11) M(4) R(8) S(2) W(16)	A(2) D(3) E(6) K(4) N(1) P(2) Q(1) T(1) Y(8)	28	8	8	88%
100	1	VHA	A(3) E(6) I(52) K(0) L(5) P(0) Q(2) T(18) V(24)	F(1) M(7) Y(1)	9	9	9	78%
101	1	RAC	D(22) N(49)	S(9)	9	2	2	100%
104	1	MDC	H(2) I(1) L(16) N(40) R(12) S(14)	A(6) D(3) E(2) F(2) K(7) M(2) Q(1) T(1) W(5) Y(2)	31	6	6	100%

105	1	TWC	F(37) Y(29)	C(1) H(2) W(11)	14	2	2	100%
106	1	GTA, TGG	V(40) W(40)		0	2	2	100%
107	1	DMC, MDG	A(9) D(1) K(11) L(6) M(3) N(7) Q(6) R(17) S(8) T(0) Y(42)	E(1) F(3) H(2) I(1)	7	12	11	92%
108	1	HDC	C(1) F(46) H(5) I(2) L(22) N(4) R(10) S(4) Y(5)	A(3) D(1) E(1) K(1) M(4) Q(1) T(1) V(1) W(7)	20	9	9	100%
109	1	DKG, WWC	F(8) G(0) I(16) L(5) M(6) N(1) R(6) V(12) W(51) Y(9)	H(1) K(4) T(1)	6	10	10	90%
110	1	SAM	D(68) E(12) H(0) Q(40)		0	4	4	75%
111	1	HDC	C(0) F(3) H(7) I(3) L(16) N(2) R(15) S(6) Y(41)	A(1) D(1) E(1) K(4) M(8) P(1) Q(5) T(1) V(3)	25	9	9	89%

*To reduce the amino acid diversity of the above table (~10^9), 'WKS' codon was removed for position 95 giving a final theoretical diversity of ~$3.3x10^8$.

Table S6: Oligonucleotides used to generate 1EBB library (synthesized by Eurofin Genomics)

**Oligo name**	**Sequence**
1ebbLib01	5′-ATTGGGAGGGGAAGACGCACWKSVWAATTGCCTTGRYARACCCGATCMDCT WCGTADMCHDCDKGSAMHDCCCGCATTTGTATGCGCCGC-3′
1ebbLib02	5′-ATTGGGAGGGGAAGACGCACWKSVWAATTGCCTTGRYARACCCGATCMDCT WCTGGDMCHDCDKGSAMHDCCCGCATTTGTATGCGCCGC -3′
1ebbLib03	5′-ATTGGGAGGGGAAGACGCACWKSVWAATTGCCTTGRYARACCCGATCMDCT WCTGGMDGHDCDKGSAMHDCCCGCATTTGTATGCGCCGC-3′
1ebbLib04	5′-ATTGGGAGGGGAAGACGCACWKSVWAATTGCCTTGRYARACCCGATCMDCT WCTGGMDGHDCWWCSAMHDCCCGCATTTGTATGCGCCGC-3′
1ebbLib05	5′-ATTGGGAGGGGAAGACGCACWKSVWAATTGCCTTGRYARACCCGATCMDCT WCTGGDMCHDCWWCSAMHDCCCGCATTTGTATGCGCCGC-3′
1ebbLib06	5′-ATTGGGAGGGGAAGACGCACWKSVWAATTGCCTTGRYARACCCGATCMDCT WCGTADMCHDCWWCSAMHDCCCGCATTTGTATGCGCCGC-3′
1ebbLib07	5′-ATTGGGAGGGGAAGACGCACWKSVWAATTGCCTTGRYARACCCGATCMDCT WCGTAMDGHDCDKGSAMHDCCCGCATTTGTATGCGCCGC -3′
1ebbLib08	5′-ATTGGGAGGGGAAGACGCACWKSVWAATTGCCTTGRYARACCCGATCMDCT WCGTAMDGHDCWWCSAMHDCCCGCATTTGTATGCGCCGC-3′

Table S7: Representative input to *SwiftLib* to design 171l directed library.

Top row indicates residue number as per 1ebb and left-most column indicates amino acid. Only mutated positions are shown in the table. Entries in each cell indicate the frequency of amino acid desired in that position. Additionally, to favor Rosetta predicted mutations, 50% weightage was placed for residues present in 1ebb-graft. For example, at position 136, no Serine was obtained from HTH-pep library sequencing but Serine was predicted by Rosetta at that position (numbering as in 171l scaffold). Subsequently, frequency of Serine was set to 40 (50% of the 80 sequences from HTH-pep library). As an optional input, up to 2 degenerate codons were allowed for each position. To penalize STOP codons, frequency was set to -10. Empty cells represent a frequency of '0'.

	135	136	137	141	142	145	146	147	148	149	150	151	152
Ala	46		2	3	40	6	40		9	3			1
Cys							1			1			
Asp	11	8			22	3			1	1		68	1
Glu	3		1	6		2			1	1		12	1
Phe	1	6		1		2	37		3	6	8		3
Gly	8		1	1		4			3	1			2
His		4				2	2		2	5	1		7
Ile	2	2	12	42		1			1	2	16		3
Lys	47		40			7			11	1	4		4
Leu	3	7	28	5		16			6	22	5		16
Met	2	9	6	7		2			3	4	6		8
Asn		3			49	40			7	4	1		2
Pro													1
Gln	3		6	2		1			6	1			5
Arg	13	3	44			12			17	10	46		15
Ser	2	40	2		9	14			8	4			6
Thr	1		2	18		1				1	1	40	41
Val	3	2	14	24				40		41	12		3
Trp	10	32	1			5	11	40		7	11		
Tyr	5	4	1	1		2	29		2	5	9		1
Stop	-10	-10	-10	-10	-10	-10	-10	-10	-10	-10	-10	-10	-10

Table S8: *SwiftLib* output with degenerate codons for 171l directed library design.

Numbering as in 171l scaffold.

Pos	Stretch	Codon	Present	Absent	Error	# Codons	# AA	%desired
135	1	DSG	A(40) G(0) R(40) S(0) T(0) W(40)	D(40)	40	6	6	50%
136	1	TSG	S(40) W(32)	D(8) F(6) H(4) I(2) L(7) M(9) N(3) R(3) V(2) Y(4)	48	2	2	100%
137	1	VDA	E(1) G(1) I(12) K(40) L(28) Q(6) R(44) V(14)	A(2) M(6) S(2) T(2) W(1) Y(1)	14	9	8	100%
141	1	RYA	A(3) I(42) T(18) V(24)	E(6) F(1) G(1) L(5) M(7) Q(2) Y(1)	23	4	4	100%
142	1	RVC	A(40) D(22) G(0) N(49) S(9) T(0)		0	6	6	67%
145	1	DBG	A(6) G(4) L(16) M(2) R(12) S(14) T(1) V(0) W(5)	D(3) E(2) F(2) H(2) I(1) K(7) Q(1) Y(2)	20	9	9	89%
146	1	GCA, TWC	A(40) F(37) Y(29)	C(1) H(2) W(11)	14	3	3	100%

147	1	GTA, TGG	V(40) W(40)		0	2	2	100%
148	1	DBG	A(6) G(4) L(16) M(2) R(12) S(14) T(1) V(0) W(5)	D(3) E(2) F(2) H(2) I(1) K(7) Q(1) Y(2)	20	9	9	89%
149	1	NDC	C(1) D(1) F(6) G(1) H(5) I(2) L(22) N(4) R(10) S(4) V(41) Y(5)	A(3) E(1) K(1) M(4) Q(1) T(1) W(7)	18	12	12	100%
150	1	DKS	C(0) F(8) G(0) I(16) L(5) M(6) R(46) S(0) V(12) W(11)	H(1) K(4) N(1) T(1) Y(9)	16	12	10	67%
151	1	ACA, GAM	D(68) E(12) T(40)		0	3	3	100%
152	1	VNS	A(1) D(1) E(1) G(2) H(7) I(3) K(4) L(16) M(8) N(2) P(1) Q(5) R(15) S(6) T(41) V(3)	F(3) Y(1)	4	24	16	100%

Table S9: Oligonucleotides used to generate 171l library (synthesized by Eurofin Genomics)

Oligo name	Sequence
171lLib01	5'-GCG GCC AAG AAC ATG GCC DSGTSGVDA TGGGCCTTG RYARVCCCCA TTDBGGCAGTA DBGNDCDKS ACAVNS CTT CAG ACG GGC ACC TGG-3'
171lLib02	5'-GCG GCC AAG AAC ATG GCC DSGTSGVDA TGGGCCTTG RYARVCCCCA TTDBGGCAGTA DBGNDCDKS GAMVNS CTT CAG ACG GGC ACC TGG-3'
171lLib03	5'- GCG GCC AAG AAC ATG GCC DSGTSGVDA TGGGCCTTG RYARVCCCCA TTDBGGCATGG DBGNDCDKS ACAVNS CTT CAG ACG GGC ACC TGG-3'
171lLib04	5'- GCG GCC AAG AAC ATG GCC DSGTSGVDA TGGGCCTTG RYARVCCCCA TTDBGGCATGG DBGNDCDKS GAMVNS CTT CAG ACG GGC ACC TGG-3'
171lLib05	5'- GCG GCC AAG AAC ATG GCC DSGTSGVDA TGGGCCTTG RYARVCCCCA TTDBGTWCGTA DBGNDCDKS ACAVNS CTT CAG ACG GGC ACC TGG-3'
171lLib06	5'- GCG GCC AAG AAC ATG GCC DSGTSGVDA TGGGCCTTG RYARVCCCCA TTDBGTWCGTA DBGNDCDKS GAMVNS CTT CAG ACG GGC ACC TGG-3'
171lLib07	5'- GCG GCC AAG AAC ATG GCC DSGTSGVDA TGGGCCTTG RYARVCCCCA TTDBGTWCTGG DBGNDCDKS ACAVNS CTT CAG ACG GGC ACC TGG-3'
171lLib08	5'- GCG GCC AAG AAC ATG GCC DSGTSGVDA TGGGCCTTG RYARVCCCCA TTDBGTWCTGG DBGNDCDKS GAMVNS CTT CAG ACG GGC ACC TGG-3'

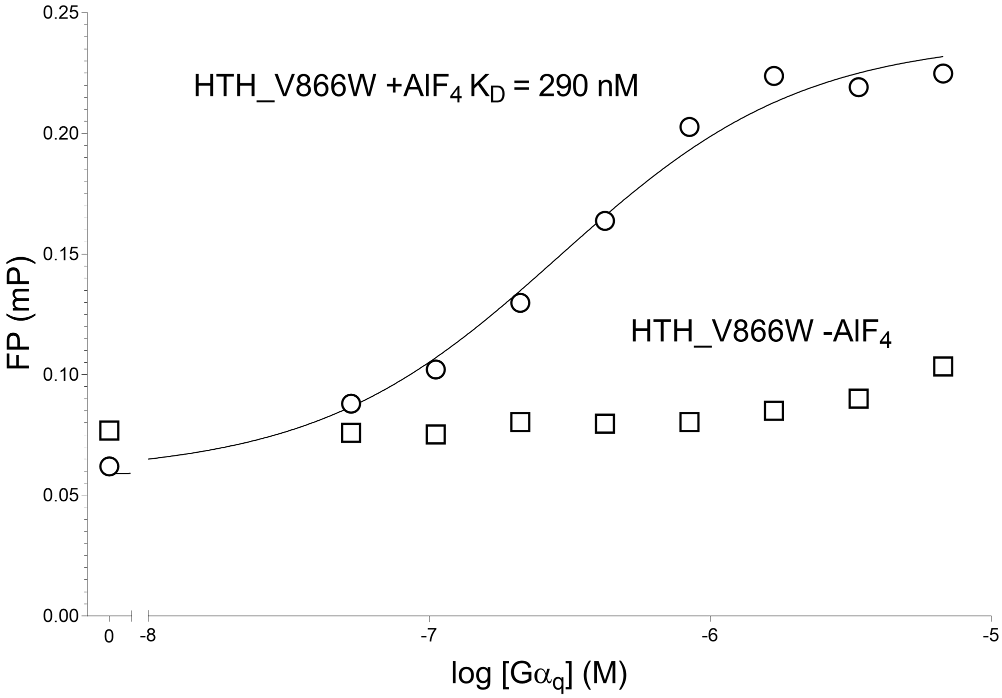

Figure S1: 21-mer HTH with the V866W mutation specifically binds the active form of $G\alpha_{q/i}$. TAMRA labeled HTH (21-mer residues 852-872) was incubated with varying concentrations of $G\alpha_{q/i}$. The experiment was performed with and without 1 mM NaF. Fluorescence polarization (mP) was measured at each concentration of $G\alpha_{q/i}$. Presented data is representative of experiment performed in triplicates.

```
Gai1                       ------MGCTLSAEDKAAVERSKMIDRNLREDGEKAAREVKLLLLGAGESGKSTIVKQMK      54
GalphaQ                    MTLESIMACCLSEEAKEARRINDEIERQLRRDKRDARRELKLLLLGTGESGKSTFIKQMR      60
GalphaQ/i-chimera          ------MGCTLSAEDKAAVERSKMIDRNLREDGEKAARELKLLLLGTGESGKSTFIKQMR      54
                                 *.* ** * * * . .. *:*:**.* ..* **:******:*******::***:

Gai1                       IIHEAGYSEEECKQYKAVVYSNTIQSIIAIIRAMGRLKIDFGDSARADDARQLFVLAGAA      114
GalphaQ                    IIHGSGYSDEDKRGFTKLVYQNIFTAMQAMIRAMDTLKIPYKYEHNKAHAQLV-REVDVE      119
GalphaQ/i-chimera          IIHGSGYSDEDKRGFTKLVYQNIFTAMQAMIRAMDTLKIPYKYEHNKAHAQLV-REVDVE      113
                           *** :***:*: : :. :**.* : :: *:****. *** :  . .  .*: :   ...

Gai1                       EEGFMTAELAGVIKRLWKDSGVQACFNRSREYQLNDSAAYYLNDLDRIAQPNYIPTQQDV      174
GalphaQ                    KVSAFENPYVDAIKSLWNDPGIQECYDRRREYQLSDSTKYYLNDLDRVADPAYLPTQQDV      179
GalphaQ/i-chimera          KVSAFENPYVDAIKSLWNDPGIQECYDRRREYQLSDSTKYYLNDLDRVADPAYLPTQQDV      173
                           : . :    ...** **:* *:* *::* *****.**: ********:*:* *:******

Gai1                       LRTRVKTTGIVETHFTFKDLHFKMFDVGGQRSERKKWIHCFEGVTAIIFCVALSDYDLVL      234
GalphaQ                    LRVRVPTTGIIEYPFDLQSVIFRMVDVGGQRSERRKWIHCFENVTSIMFLVALSEYDQVL      239
GalphaQ/i-chimera          LRVRVPTTGIIEYPFTFKDLHFRMVDVGGQRSERRKWIHCFEgVTaIiFcVALSEYDQVL      233
                           **.** ****:*  * ::.: *:*.*********:*******.**:*:* ****:** **

Gai1                       AEDEEMNRMHESMKLFDSICNNKWFTDTSIILFLNKKDLFEEKIKKSPLTICYPEYAGSN      294
GalphaQ                    VESDNENRMEESKALFRTIITYPWFQNSSVILFLNKKDLLEEKIMYSHLVDYFPEYDGPQ      299
GalphaQ/i-chimera          VESDNENRMEESKALFRTIITYPWFQNSSVILFLNKKDLLEEKIMYSHLVDYFPEYDGPQ      293
                           .*.:: ***.**  ** :* .  ** ::*:*********:****  * *.  :*** * :

Gai1                       TYEEAA-AYIQCQFEDLNKRKDTKEIYTHFTCATDTKNVQFVFDAVTDVIIKNNLKDCGL      353
GalphaQ                    RDAQAAREFILKMFVDLNPD-SDKIIYSHFTCATDTENIRFVFAAVKDTILQLNLKEYNL      358
GalphaQ/i-chimera          RDAQAAREFILKMFVDLNPD-SDKIIYSHFTCATDTENIRFVFAAVKDTILQLNLKEYNL      352
                              :**  :*   * ***   . * **:********:*::*** **.*.*:: ***: .*

Gai1                       F        354
GalphaQ                    V        359
GalphaQ/i-chimera          V        353
                           .
```

Figure S2: Sequence alignment of Gα_q, Gα_{i1} and G$\alpha_{q/i}$.
Figure was generated by ClustalOmega online tool.

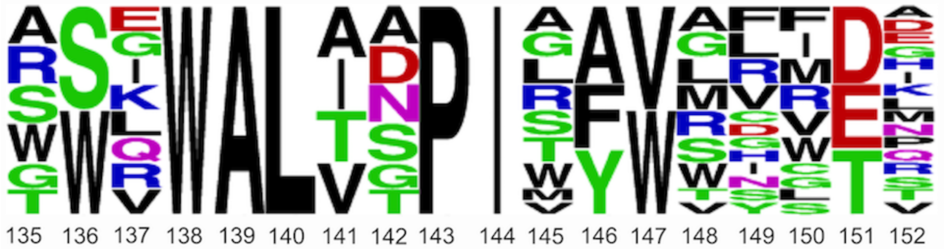

Figure S3: Design of 171l directed library.

A subset of amino acids for each given position as outlined by an amino acid consensus profile generated by Web Logo is shown here. Theoretical diversity of the library was estimated to be ~$6x10^{9}$.

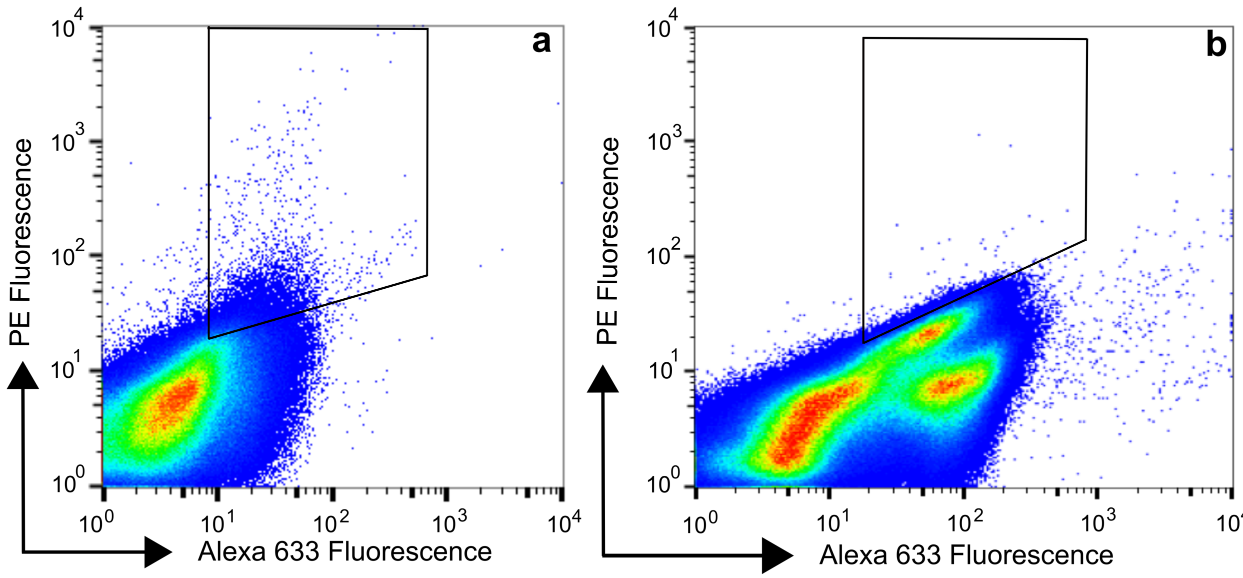

Figure S4: FACS analysis of 1ebb yeast library.

(a) Yeast cells displaying 1ebb mutants were simultaneously labeled with a chicken anti-cmyc antibody and 1 μM biotinylated Gα_q followed by secondary labeling with a goat anti-chicken antibody conjugated to Alexa Fluor 633 (to detect expression) and Streptavidin conjugated to PE (to detect binding) and analyzed by flow cytometry. Cells in the polygon region were sorted from round 1, grown and further sorted by labeling at successively lower concentrations of Gα_q and other reagents as described before until labeling with 4 nM Gα_q at sort 7 as shown in **(b)**. Yeast cells from round 7 were plated to isolate individual clones for further analysis.

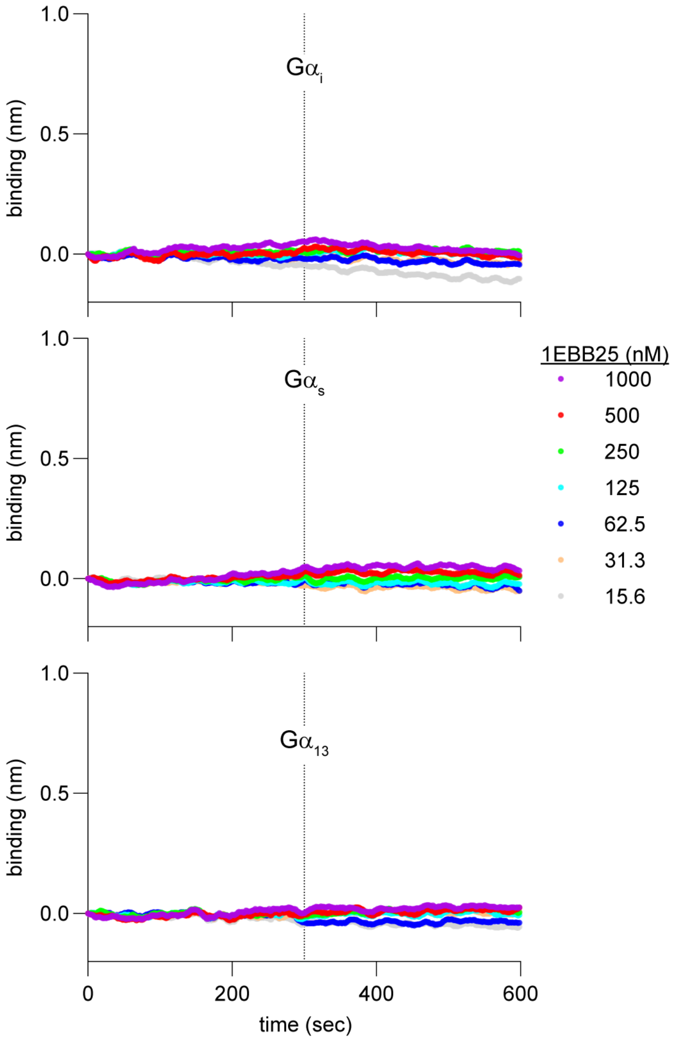

Figure S5: BLI experiment testing binding of 1EBB25 to other major Gα protein: Gα i, Gα s, and Gα 13/i.

1EBB25 does not bind other major Gα proteins. BLI streptavidin sensors were loaded with .025 mg/mL biotinylated Gα protein. Sensors were incubated in different concentrations of 1EBB25 in the presence of AlF_4 for 5 minutes, and then in buffer without 1EBB25.

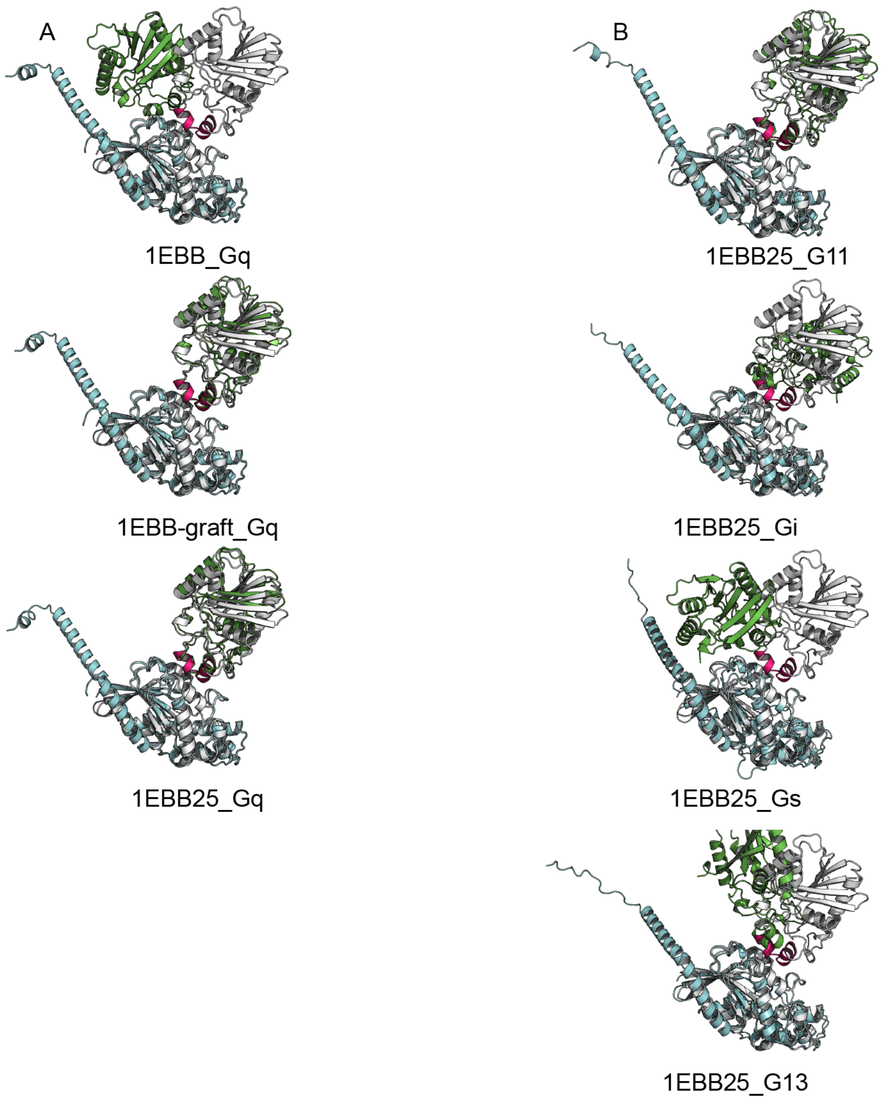

Figure S6: AlphaFold predictions recapitulate Rosetta model

AlphaFold predictions (shown in green) were made using the ColabFold framework (Mirdita, Ovchinnikov, and Steinegger, *biorxiv* 2021). Rosetta model of 1EBB-graft bound to $G\alpha_q$ is colored as white cartoon. HTH motif is colored as hot pink. **A)** AlphaFold predicts 1EBB-graft and 1EBB25 to bind $G\alpha_q$ in the same orientation as the Rosetta model, but not 1EBB. **B)** AlphaFold predicts 1EBB25 to bind $G\alpha_{11}$ in the same orientation as $G\alpha_q$. Expectedly, this orientation is not predicted against other G proteins: $G\alpha_i$, $G\alpha_s$, and $G\alpha_{13}$.

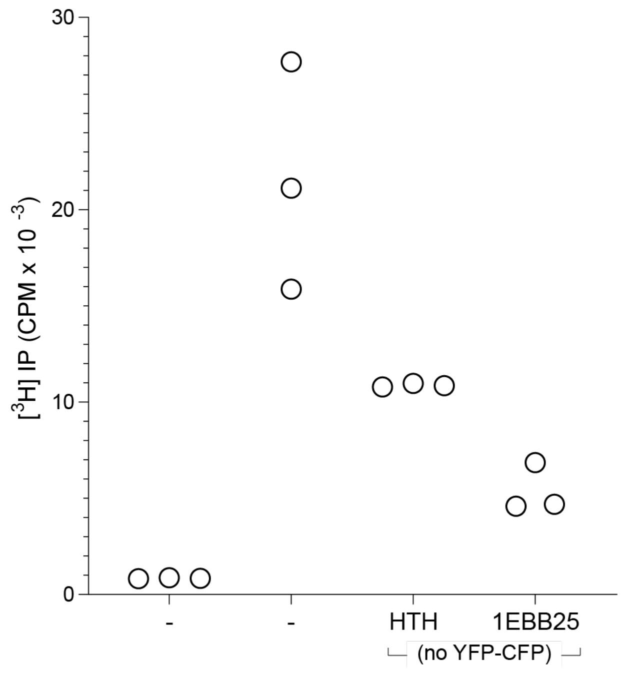

Figure S7: Inhibition of $G\alpha_q$_Q209L by HTH and 1EBB25 without YFP and CFP

1EBB25 still efficaciously inhibits $G\alpha_q$_Q209L without YFP-CFP sandwich. [^{3}H] IP production from HEK293 cells was quantified after co-transfecting G protein and inhibitor. Both inhibitors in this experiment were expressed with an N-terminal Myc tag and C-terminal Rac1 CaaX box.

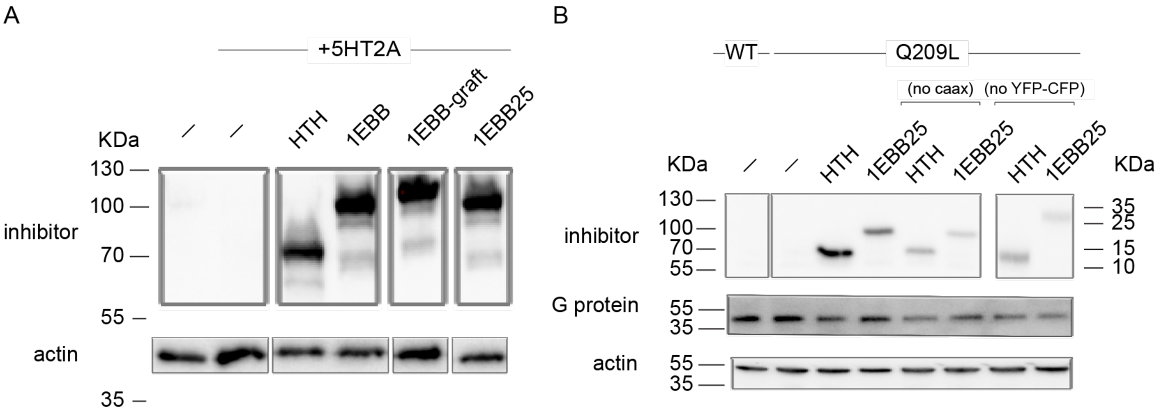

Figure S8: Western blots of co-transfection experiments

Efficacy of inhibition by 1EBB25 is not due to changes in protein expression. Unless otherwise noted, all inhibitor constructs are sandwiched between YFP and CFP, and contain a Rac1 CAAX box as a C-terminal tail. All inhibitors also contain an N-terminal Myc tag. All G protein constructs contain an N-terminal HA tag. **A)** 1EBB25 expresses to the same degree as HTH. Expression of constructs when testing receptor mediated $G\alpha_q$ activity. HEK293 cells are co-transfected with 2 plasmids expressing 5HT2A receptor or inhibitor. A total of 300ng of DNA was transfected: 100 ng 5HT2A, 100 ng inhibitor, and 100 ng of empty plasmid. No inhibitor indicates an empty plasmid without a gene insert. **B)** Expression of inhibitor constructs does not significantly change expression of the G protein. Expression of constructs when testing oncogenic $G\alpha_q$ Q209L. A total of 300 ng of DNA was transfected: 10 ng G protein, 20 ng inhibitor, 270 ng empty plasmid.

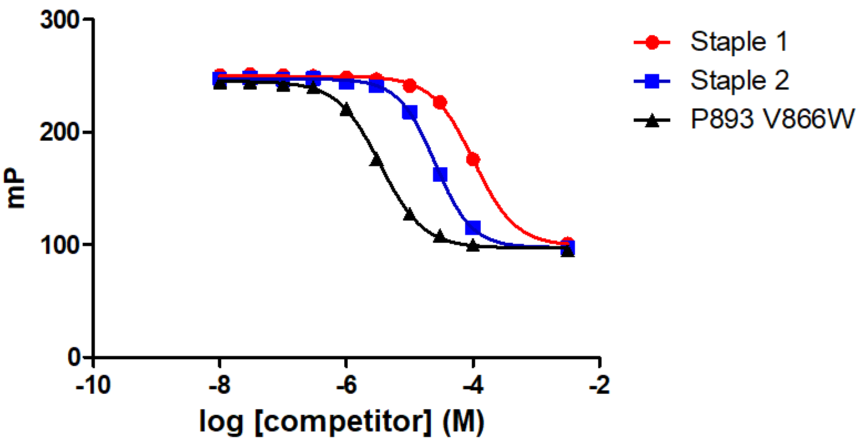

Figure S9 Competitive fluorescence polarization assay was performed similar to Fig. 5. Stapled helices do not improve affinity for $G\alpha_{q/i}$. Briefly, 800 nM $G\alpha_{q/i}$ was incubated with 200 nM TAMRA-HTH_V866W. Varying concentrations of the indicated competitor, competed with TAMRA labeled peptide. Staple 1 has a staple between residues D854 and A858. Staple 2 has a staple between residues K864 and L868. P893 is the HTH peptide with the V866W mutation and no TAMRA label.
